# Potential for additional government spending on HIV/AIDS in 137 low-income and middle-income countries: an economic modelling study

**DOI:** 10.1016/S2352-3018(19)30038-4

**Published:** 2019-04-26

**Authors:** Annie Haakenstad, Mark W Moses, Tianchan Tao, Golsum Tsakalos, Bianca Zlavog, Jennifer Kates, Adam Wexler, Christopher J L Murray, Joseph L Dieleman

**Affiliations:** a Harvard T H Chan School of Public Health, Harvard University, Boston, MA, USA; b Institute for Health Metrics and Evaluation, University of Washington, Seattle, WA, USA; c Kaiser Family Foundation, San Francisco, CA, USA

## Abstract

**Background:**

Between 2012 and 2016, development assistance for HIV/AIDS decreased by 20·0%; domestic financing is therefore critical to sustaining the response to HIV/AIDS. To understand whether domestic resources could fill the financing gaps created by declines in development assistance, we aimed to track spending on HIV/AIDS and estimated the potential for governments to devote additional domestic funds to HIV/AIDS.

**Methods:**

We extracted 8589 datapoints reporting spending on HIV/AIDS. We used spatiotemporal Gaussian process regression to estimate a complete time series of spending by domestic sources (government, prepaid private, and out-of-pocket) and spending category (prevention, and care and treatment) from 2000 to 2016 for 137 low-income and middle-income countries (LMICs). Development assistance data for HIV/AIDS were from Financing Global Health 2018, and HIV/AIDS prevalence, incidence, and mortality were from the Global Burden of Disease study 2017. We used stochastic frontier analysis to estimate potential additional government spending on HIV/AIDS, which was conditional on the current government health budget and other finance, economic, and contextual factors associated with HIV/AIDS spending. All spending estimates were reported in 2018 US$.

**Findings:**

Between 2000 and 2016, total spending on HIV/AIDS in LMICs increased from $4·0 billion (95% uncertainty interval 2·9–6·0) to $19·9 billion (15·8–26·3), spending on HIV/AIDS prevention increased from $596 million (258 million to 1·3 billion) to $3·0 billion (1·5–5·8), and spending on HIV/AIDS care and treatment increased from $1·1 billion (458·1 million to 2·2 billion) to $7·2 billion (4·3–11·8). Over this time period, the share of resources sourced from development assistance increased from 33·2% (21·3–45·0) to 46·0% (34·2–57·0). Care and treatment spending per year on antiretroviral therapy varied across countries, with an IQR of $284–2915. An additional $12·1 billion (8·4–17·5) globally could be mobilised by governments of LMICs to finance the response to HIV/AIDS. Most of these potential resources are concentrated in ten middle-income countries (Argentina, China, Colombia, India, Indonesia, Mexico, Nigeria, Russia, South Africa, and Vietnam).

**Interpretation:**

Some governments could mobilise more domestic resources to fight HIV/AIDS, which could free up additional development assistance for many countries without this ability, including many low-income, high-prevalence countries. However, a large gap exists between available financing and the funding needed to achieve global HIV/AIDS goals, and sustained and coordinated effort across international and domestic development partners is required to end AIDS by 2030.

**Funding:**

The Bill & Melinda Gates Foundation.

## Introduction

Although HIV/AIDS mortality and incidence have declined by almost 50% since 2000, the HIV/AIDS agenda is far from realised. In 2016, more than 1 million people died from HIV/AIDS and nearly 2 million people were infected with HIV.^1^, ^2^ A sustained commitment to addressing HIV/AIDS is crucial for attaining the ambitious goal of ending AIDS by 2030 set by UNAIDS. A cornerstone of this goal is the 90-90-90 targets: ensure 90% of people living with HIV/AIDS know their status, 90% of those diagnosed with HIV/AIDS receive antiretroviral therapy (ART), and achieve viral suppression in 90% of patients on ART by the year 2020.^3^, ^4^ Effective and appropriate care and treatment for HIV/AIDS not only reduces mortality but slows transmission of the virus, which is crucial to ending AIDS by 2030.^5^, ^6^

Despite ambitious HIV/AIDS targets, investments in the fight against HIV/AIDS have waned. Between 2012 and 2016, development assistance for HIV/AIDS dropped by 20·0%,^7^ resulting in declines in total HIV/AIDS financing in low-income countries, where external funding constitutes 85% of all HIV/AIDS spending.^7^ Domestic financing is thus critical to the fight against HIV/AIDS, but little is known about whether countries, and more specifically governments, can mobilise enough funding to fill the financing gaps left by donors.

**Research in context**

**Evidence before this study**

In 2018, the Institute for Health Metrics and Evaluation published a complete time series of HIV/AIDS spending estimates by source and function. These estimates showed that development assistance for HIV/AIDS has declined since 2012, while domestic spending on HIV/AIDS continued to grow. In 2018, UNAIDS reported domestic spending and, with the Kaiser Family Foundation, published estimates of development assistance for HIV/AIDS for select development assistance partners. Previous analyses have examined the potential for additional HIV/AIDS spending by either comparing spending levels to established benchmarks or using regression-based methods. Studies that use a benchmarking perspective do so by calculating potential spending on the basis of the difference between current HIV/AIDS spending and benchmarks or norms, such as the Abuja target of dedicating 15% of government budgets to health, donor preferences, and targets for revenue, debt, and gross domestic product per capita. Studies that used regression-based methods have estimated the potential for additional spending by comparing the difference between actual and predicted spending.

**Added value of this study**

Our study updates previously published estimates of HIV/AIDS expenditure by incorporating additional HIV/AIDS spending data and expanding the time frame of analysis to include 2016. We used a stochastic frontier analysis model to estimate the potential for additional government spending on HIV/AIDS, conditional on the current government health budget and other public finance, economic, and contextual factors. Compared with previous studies, this study uses a regression-based approach that explicitly benchmarks countries to one another to measure the potential for governments to spend more on HIV/AIDS, relative to the country's fixed health budget and other public finance, economic, and contextual factors. Using estimates of spending and potential spending, we assess how countries are progressing in meeting the necessary funding targets to achieve the UNAIDS goal of ending the HIV/AIDS epidemic.

**Implications of all the available evidence**

Domestic and international resources fund HIV/AIDS prevention, and the care and treatment of people living with HIV/AIDS in low-income and middle-income countries (LMICs). However, many countries remain reliant on development assistance: nearly 17 million people living with HIV/AIDS live in countries where development assistance finances over 75% of the HIV/AIDS care and treatment budget. We estimate that governments of LMICs have the ability to spend an additional US$12·1 billion (95% uncertainty interval 8·4–17·5) per year on HIV/AIDS, but this figure masks substantial variation. Over 80% of the potential additional government resources are estimated to come from 10 middle-income countries (Argentina, China, Colombia, India, Indonesia, Mexico, Nigeria, Russia, South Africa, and Vietnam). LMICs also cannot fill in the potential financing void left by a decline in development assistance. The reallocation of resources away from countries capable of achieving UNAIDS funding targets with the potential government resources could free up about $1 billion in additional funding, but these funds would fall short of the nearly $5·5 billion in additional resources required to fund the UNAIDS fast-track approach. Although governments must play a critical part in HIV/AIDS financing going forward, the achievement of global goals in the fight against HIV/AIDS will continue to require substantial financial resources from donors.

With the ambitious global targets and declines in development assistance in mind, we expanded previously published efforts to track HIV/AIDS spending by financing source and spending category and estimated the amount of additional spending governments could mobilise for HIV/AIDS. This analysis aimed to support the global HIV/AIDS community in measuring spending on HIV/AIDS and the global potential for additional government spending on HIV/AIDS, with the intention of informing future resource needs and the distributions of development assistance to support progress towards the global goal of ending AIDS by 2030.

## Methods

### Estimation of HIV/AIDS spending by financing source and function

The data and methods used to estimate HIV/AIDS spending have been described in depth previously.^7^ We briefly describe these methods and report updates here. We extracted spending data from five main sources: the AIDSinfo online database published by UNAIDS, Global Fund concept notes and proposals, National Health Accounts, National AIDS Spending Assessments, and the AIDS data hub. With these data, we used spatiotemporal Gaussian process regression (ST-GPR) to predict spending for five HIV/AIDS domestic financing sources (domestic, government, private, prepaid private, and out-of-pocket financing) and three HIV/AIDS domestic spending categories (prevention, care and treatment, and other spending) from 2000 to 2016. Other spending is spending on HIV/AIDS programming that is not focused on care and treatment or prevention, such as spending on health system strengthening. Estimates of financing source were estimated as a fraction of the associated all-health spending estimates from published literature.^8^ For example, HIV/AIDS domestic spending was estimated as a fracti on of total domestic health spending. We used a multistep aggregation and scaling procedure across all spending models to ensure government, prepaid private, and out-of-pocket spending would sum to total domestic spending. Total HIV/AIDS spending and total HIV/AIDS spending by spending category were calculated by adding estimates of development assistance from Financing Global Health 2018^8^ to the estimates of domestic spending on HIV/AIDS.

Covariates in the ST-GPR models were all sourced from the Global Burden of Disease (GBD) study 2017. The specific covariates we used varied based on the model but broadly included ART coverage, the natural log of 10 year lag-distributed gross domestic product (LDI) per capita, the natural log of HIV prevalence, the natural log of HIV incidence, the natural log of the HIV mortality rate, and the natural log of ART prices.^1^, ^2^ LDI is widely used in the estimation of health outcomes and attempts to account for accrued wealth by taking the weighted average of gross domestic product (GDP) over time, with more weight given to more recent years. The selected covariates were used in the first step of ST-GPR, a linear mixed-effects regression with random effects on GBD region and super-region. Additional details on the modelling process are in the appendix (p 8), including the covariates used in each of the models and a description of currency conversion and deflation.

We improved upon published spending estimates in three ways. First, an additional 3204 datapoints were added to the 5385 previously used to estimate HIV/AIDS expenditure.^7^ Most of these new datapoints are from the most recent UNAIDS HIV Financial Dashboard. The remaining datapoints are from National AIDS Spending Assessments and Global AIDS Response Progress Reports released in November, 2018. Second, all spending variables were converted into 2018 US$ to harmonise results with other published estimates of global health resources^3^, ^4^, ^9^, ^10^ and to provide more tangible estimates for international and national policy makers. Third, the administrative costs of running development assistance projects globally were excluded when we report spending by region or country, because these resources were not spent within a low-income and middle-income country (LMIC) and typically capture spending on global activities or project administration. Exclusion of these administrative costs also improves alignment with the definition of health spending used by the Systems of Health Accounts.^11^

We focused on the 137 countries classified as low or middle income by the World Bank in 2017. Estimates of HIV/AIDS spending reported by GBD super-region and World Bank income group were aggregated and divided by the aggregates of the population. Reported spending per person or person living with HIV/AIDS thus reflects the region or income group as a whole, rather than the average at the country level, to avoid averages where countries with vastly different population sizes count equally. Care and treatment spending was also reported by dividing the total spending on care and treatment by the total number of individuals on ART (the product of population, HIV/AIDS prevalence, and ART coverage) to reflect the HIV/AIDS population that has linked to care. Total prevention spending was divided by the number of prevalent cases (the product of population and HIV prevalence), to represent how countries' prevention spending responds to the size of the population that can transmit HIV.

### Potential for additional government HIV/AIDS spending

We used stochastic frontier analysis^12^, ^13^ to estimate the potential for governments to spend additional resources on HIV/AIDS, relative to their fixed health budget and other public finance, economic, and contextual factors. Stochastic frontier analysis is an econometric regression method used by economists to estimate how well entities such as factories, hospitals, or countries produce outputs with a given set of inputs, by benchmarking each entity to other entities. We used this approach to assess how well countries produced government HIV/AIDS spending, controlling for factors such as the HIV/AIDS burden and contextual financing indicators. This analysis measures how well one country does against another by estimating an efficiency score on an interval from 0 to 1, where an estimated efficiency score closer to 1 showed that a government was close to its maximum potential to spend additional resources on HIV/AIDS and an efficiency score closer to zero showed substantial capacity to increase government HIV/AIDS spending.

Our stochastic frontier analysis model used a 2016 cross-section of data and regressed government spending on HIV/AIDS per capita on the following covariates: HIV/AIDS prevalence, mortality, incidence, total domestic health spending per capita net domestic HIV spending per capita, general government spending per capita net domestic health spending per capita,^8^ LDI per capita, the Healthcare Access and Quality Index,^14^ and every possible interaction between the covariates, each covariate squared, and GBD super-region dummies. The Healthcare Access and Quality Index is a measure of each country's health-care quality and access derived from the mortality rates of amenable causes of death. We chose stochastic frontier analysis because it benchmarks countries against each other, based on key included covariates, to estimate the maximum production of resources with a given set of inputs. Similar to previous studies^15^, ^16^, ^17^ estimating potential spending on HIV/AIDS, we believe government spending on HIV/AIDS is dependent on the income (proxied by LDI per capita) and needs (proxied by HIV/AIDS prevalence, mortality and incidence) of the country. Additionally, we believe that such spending is dependent on the available level of general government expenditure and the level of government health spending. Critically, we do not incorporate any measure of government corruption^15^ or programme mismanagement because this effect is captured in the efficiency term. Incorporating the Healthcare Access and Quality Index assumes that countries maintain the current level of quality and do not sacrifice quality to generate more resources for HIV/AIDS.

We took the natural log of all covariates (before they were interacted or squared). The squared and the interacted terms were included in the regression to increase its flexibility. The inclusion of these terms resulted in the lowest Bayesian information out of all model specifications tested (appendix p 11).

To estimate the potential for countries to spend more resources on HIV/AIDS in 2016, at the country level, we divided our estimate of government spending on HIV/AIDS per capita by our estimate of a country's efficiency of producing HIV/AIDS spending, to represent a government's maximum potential spending based on the results of the stochastic frontier analysis model. We subtracted observed government spending on HIV/AIDS per capita and multiplied the quantity by the total population. We used the following calculation:

Additional Gov't HIV/AIDS=(Gov'tHIV/AIDScapθ-Gov'tHIV/AIDScap)⋅Population

where a country's estimated government spending on HIV/AIDS per capita is denoted by Gov't HIV/AIDS_cap_, while the estimated efficiency of a country in producing government spending on HIV/AIDS is denoted by θ and Additional Gov't HIV/AIDS represents the additional funds a government could devote to HIV/AIDS. Population was the population of each country sourced from GBD 2017.^16^

### Propagating uncertainty

We captured both model and data uncertainty. The GBD 2017 study^16^ developed ST-GPR to capture data and model uncertainty using a Gaussian process regression. This feature allowed us to generate 1000 draws of the posterior distributions of the HIV/AIDS spending models, which we used during the process of aggregating and raking to propagate uncertainty to our final estimates of HIV/AIDS spending. In estimating potential government spending on HIV/AIDS, we estimated our stochastic frontier analysis regression and generated 1000 draws of the posterior distribution of the efficiency estimates. We calculated additional government spending using the draws of efficiency and government spending on HIV/AIDS per capita. The 95% uncertainty intervals (UIs) of all estimates were calculated by taking the 2·5th and 97·5th percentiles of the draws.

### Role of the funding source

The funder of the study had no role in study design, data collection, data analysis, data interpretation, or writing of the report. All authors had full access to all the data in the study. The corresponding author had final responsibility for the decision to submit for publication.

## Results

**Figure 1:**
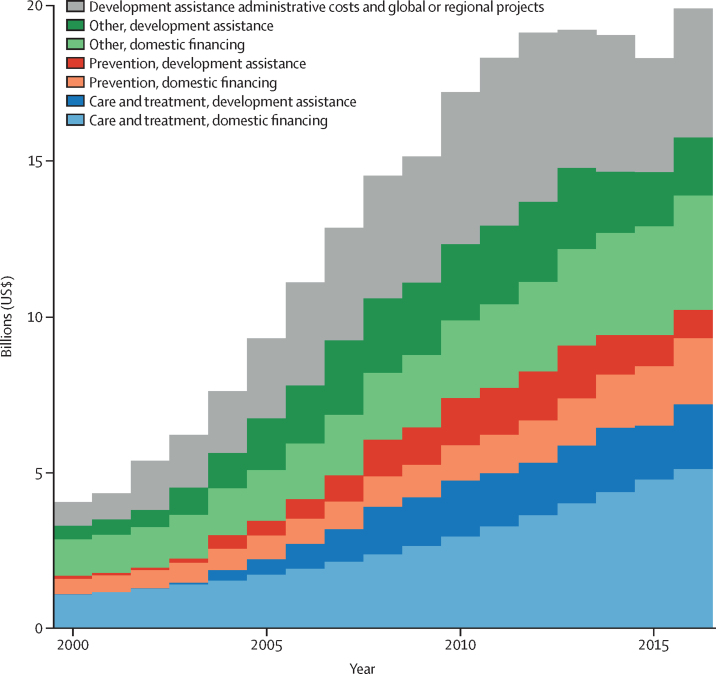
HIV/AIDS spending by financing source and spending category over time, 2000–16 All spending estimates are in 2018 US$.

Between 2000 and 2016, total spending on HIV/AIDS increased from $4·0 billion (2·9–6·0) to $19·9 billion (15·8–26·3) in LMICs (figure 1). The resource sourced from development assistance increased from increased from 33·2% (21·3–45·0) in 2000 to 46·0% (34·2–57·0) in 2016.

Total HIV/AIDS care and treatment spending increased from $1·1 billion (458·1 million to 2·2 billion) in 2000 to $7·2 billion (4·3–11·8) in 2016, financing access to ART for 16·4 million HIV/AIDS patients in LMICs. The domestic fraction of total care and treatment spending decreased from 99·6% (99·2–99·8) of care and treatment spending in 2000 to 69·0% (51·2–82·3) in 2016. Between 2010 and 2016, domestic spending in this area continued to grow by 9·7% (9·4–9·9) annually, as development assistance for care and treatment increased by 2·5% per year.

**Figure 2:**
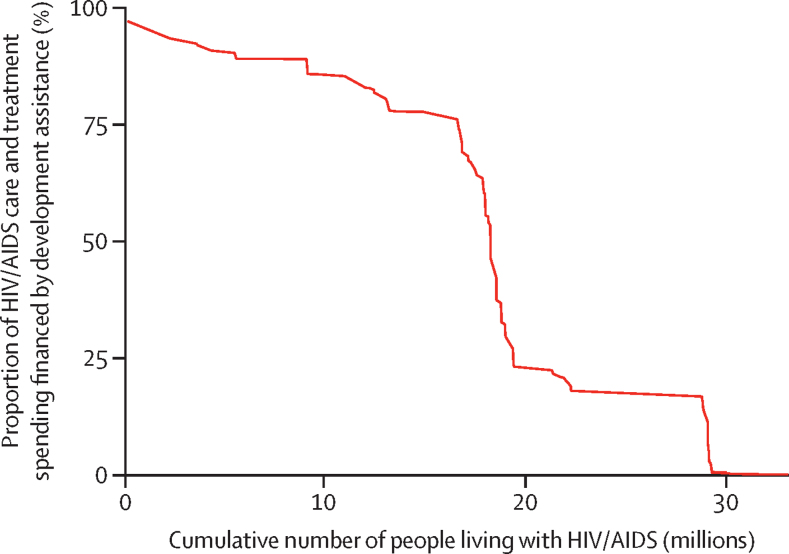
Population living with HIV/AIDS and proportion of care and treatment spending financed by development assistance, 2016

Financing of care and treatment of HIV/AIDS is largely dependent on development assistance: 33·0 million (92·2%) of 35·8 million people living with HIV/AIDS live in countries that receive development assistance for HIV/AIDS. 18·3 million people with HIV/AIDS, which is 56·0% of all people living with HIV in LMICs, live in countries where development assistance finances over 50% of the care and treatment budget (figure 2). 16·6 million people with HIV/AIDS live in 23 countries where development assistance finances over 75% of the care and treatment; of these countries, 21 are in sub-Saharan Africa (the exceptions are Haiti and Myanmar). Haiti is the most reliant on development assistance for health, which finances 96·9% of total HIV/AIDS care and treatment spending (table).

**Table:** Total spending on HIV/AIDS by spending category and financing source, and total government spending and potential, additional government spending on HIV/AIDS, 2016

	**Total spend on HIV/AIDS in millions ($)**	**Proportion of care and treatment spend out of total spend on HIV/AIDS (%)**	**Proportion of prevention spend out of total spend on HIV/AIDS (%)**	**Proportion of spend on care and treatment from development assistance for health out of care and treatment spend on HIV/AIDS (%)**	**Proportion of spend on prevention from development assistance for health out of prevention spend on HIV/AIDS (%)**	**Total government spend on HIV/AIDS in millions ($)**	**Potential additional government spend on HIV/AIDS in millions ($)**
**World Bank income group**							
Low	2738·0 (2525·0–3102·0)	41·4% (40·6–42·9)	23·2% (20·7–26·8)	86·3% (73·1–94·9)	74·3% (56·2–89·2)	307·7 (155·6–547·8)	390·5 (205·2–685·9)
Lower middle	3435·0 (2824·3–4423·2)	37·5% (34·1–43·3)	24·7% (17·7–33·7)	65·2% (42·8–85·2)	43·0% (22·8–68·0)	1221·1 (754·9–1896·9)	1594·4 (947·2–2576·6)
Upper middle	9566·4 (6293·9–14 659·7)	48·4% (36·4–58·5)	14·8% (8·3–23·8)	6·9% (3·4–12·8)	8·7% (3·0–19·6)	8180·0 (5376·3–11 956·4)	10 158·1 (7225·1–14 234·0)
**GBD super-region**							
Central Europe, eastern Europe, and central Asia	890·0 (582·4–1351·1)	39·8% (32·0–46·2)	20·9% (11·8–30·3)	6·7% (3·5–11·9)	14·3% (5·5–33·1)	759·5 (458·6–1213·5)	896·2 (514·8–1483·5)
Latin America and Caribbean	4184·6 (2733·8–6545·0)	55·2% (41·3–64·8)	10·8% (4·8–22·2)	3·8% (1·9–7·1)	8·3% (1·9–21·2)	3377·5 (2298·7–4649·8)	1107·6 (728·6–1650·0)
North Africa and Middle East	388·8 (235·6–633·5)	34·6% (27·1–41·9)	48·4% (36·9–56·4)	4·6% (2·1–8·8)	6·9% (3·3–13·5)	343·7 (197·7–569·6)	40·1 (24·0–63·5)
South Asia	533·8 (383·8–762·9)	13·9% (6·7–30·0)	39·2% (18·5–65·0)	34·2% (8·4–74·3)	10·5% (3·3–22·8)	353·4 (232·3–531·9)	387·1 (254·7–579·6)
Southeast Asia, east Asia, and Oceania	2014·3 (1604·5–2537·0)	26·6% (15·1–42·1)	25·5% (13·7–40·2)	12·6% (5·6–24·7)	12·1% (5·4–24·8)	1701·1 (1304·7–2205·6)	7368·5 (5578·6–9712·5)
Sub-Saharan Africa	7298·0 (5804·6–9762·6)	47·8% (43·5–52·6)	18·6% (16·7–21·1)	56·0% (37·0–75·3)	59·1% (37·6–80·2)	2746·3 (1498·9–4641·3)	2040·7 (1067·2–3589·8)
**Country**							
Afghanistan	6·6 (5·6–8·8)	26·0% (20·6–34·4)	36·5% (34·5–39·3)	49·0% (26·6–69·6)	74·1% (50·5–90·5)	0·6 (0·2–0·9)	1·3 (0·5–2·2)
Albania	1·7 (1·2–2·3)	71·8% (66·8–74·7)	2·1% (0·3–7·9)	0·0% (0·0–0·0)	0·0% (0·0–0·0)	1·6 (1·1–2·2)	2·7 (1·9–3·8)
Algeria	19·0 (13·2–29·5)	87·0% (83·7–88·7)	9·3% (3·3–18·8)	0·0% (0·0–0·0)	0·1% (0·0–0·2)	18·8 (13·0–29·3)	0·0 (0·0–0·0)
American Samoa	0·3 (0·2–0·4)	41·7% (13·9–59·3)	20·5% (7·9–38·7)	0·0% (0·0–0·0)	0·0% (0·0–0·0)	0·3 (0·2–0·4)	0·0 (0·0–0·0)
Angola	97·4 (73·1–128·2)	35·0% (24·2–52·5)	24·4% (15·6–32·6)	47·7% (21·7–83·7)	14·8% (7·7–28·4)	67·6 (43·2–98·4)	0·0 (0·0–0·0)
Argentina	430·0 (298·5–592·9)	39·5% (36·6–42·6)	0·9% (0·4–1·9)	0·0% (0·0–0·0)	1·0% (0·3–2·6)	427·4 (296·0–589·4)	302·8 (209·7–417·5)
Armenia	5·7 (4·7–7·1)	32·1% (30·5–33·5)	25·9% (24·3–31·0)	55·8% (42·0–69·2)	79·4% (52·0–97·1)	2·6 (1·7–4·1)	3·2 (2·1–4·9)
Azerbaijan	11·3 (8·1–16·4)	17·7% (14·1–22·6)	56·2% (55·2–57·1)	42·3% (21·7–68·8)	43·2% (28·9–58·3)	6·9 (3·7–11·9)	3·4 (1·8–5·8)
Bangladesh	13·1 (10·9–15·8)	17·4% (11·8–34·5)	29·2% (12·8–50·7)	59·0% (21·7–92·0)	30·8% (11·9–67·9)	5·8 (3·9–8·5)	7·5 (5·0–11·0)
Belarus	20·6 (15·6–26·4)	13·6% (9·3–19·8)	62·6% (58·6–65·6)	26·2% (12·8–46·4)	15·3% (11·1–21·2)	15·1 (10·7–20·6)	0·0 (0·0–0·0)
Belize	2·1 (1·5–3·1)	15·3% (7·7–25·1)	54·6% (42·8–60·3)	18·2% (6·3–41·6)	2·7% (1·6–4·5)	1·7 (1·1–2·5)	0·9 (0·6–1·4)
Benin	21·3 (19·2–24·4)	26·9% (25·1–29·7)	32·9% (28·8–37·2)	80·4% (62·9–94·3)	70·2% (53·5–87·8)	4·7 (2·7–7·7)	1·8 (1·0–2·9)
Bhutan	1·7 (1·5–2·2)	29·4% (20·2–41·4)	21·3% (10·2–37·4)	54·7% (28·5–85·7)	38·0% (14·3–77·6)	0·8 (0·5–1·2)	0·0 (0·0–0·0)
Bolivia	13·4 (9·0–20·5)	47·2% (37·0–54·8)	24·1% (19·5–30·6)	28·2% (15·0–49·5)	28·7% (13·8–48·5)	8·5 (4·7–14·3)	15·7 (8·6–26·4)
Bosnia and Herzegovina	5·0 (3·6–7·0)	68·5% (62·0–72·1)	4·3% (3·2–8·6)	3·3% (2·2–4·9)	58·3% (17·9–92·6)	4·4 (3·0–6·4)	0·0 (0·0–0·0)
Botswana	254·9 (176·6–363·2)	41·1% (33·0–45·2)	7·5% (5·7–10·7)	28·7% (17·9–49·3)	30·6% (13·7–53·0)	196·0 (124·7–279·5)	0·0 (0·0–0·0)
Brazil	2532·5 (1544·6–4250·3)	61·7% (44·9–71·0)	8·1% (1·1–22·6)	0·0% (0·0–0·0)	1·9% (0·2–8·8)	2163·6 (1490·5–2863·7)	0·0 (0·0–0·0)
Bulgaria	8·2 (5·9–11·4)	40·3% (32·7–45·1)	3·7% (3·0–6·5)	10·3% (6·3–16·8)	64·9% (23·7–93·7)	7·0 (4·8–10·2)	11·0 (7·4–15·9)
Burkina Faso	30·5 (21·7–44·6)	33·1% (27·8–38·2)	37·1% (31·3–42·2)	34·8% (19·8–55·0)	36·3% (20·7–57·3)	12·3 (5·0–25·2)	0·0 (0·0–0·0)
Burundi	26·0 (24·6–28·5)	28·2% (28·0–28·6)	21·8% (20·6–23·5)	89·0% (79·6–94·7)	80·8% (68·0–90·0)	2·4 (1·0–4·9)	1·4 (0·6–3·0)
Cambodia	33·5 (30·3–38·1)	23·0% (20·9–25·6)	23·2% (20·6–27·2)	74·8% (58·6–90·2)	62·0% (46·1–76·3)	7·1 (4·2–11·1)	45·3 (26·7–70·1)
Cameroon	67·4 (58·6–84·4)	55·0% (54·0–55·6)	18·3% (17·5–18·8)	80·9% (65·8–91·3)	83·9% (69·7–93·2)	12·3 (5·2–26·0)	28·2 (11·9–59·5)
Cape Verde	1·4 (1·0–2·1)	43·2% (29·2–55·7)	13·3% (4·1–26·3)	0·0% (0·0–0·0)	0·0% (0·0–0·0)	1·3 (0·9–2·0)	0·7 (0·5–1·0)
Central African Republic	10·4 (10·1–11·0)	6·6% (4·8–9·5)	5·5% (4·9–6·7)	66·8% (42·2–90·4)	80·9% (62·2–92·5)	0·6 (0·4–0·9)	0·3 (0·2–0·4)
Chad	15·3 (12·6–20·5)	30·6% (27·0–33·3)	33·1% (29·7–36·3)	56·9% (38·1–76·6)	56·8% (37·7–75·0)	6·0 (3·3–10·9)	0·0 (0·0–0·0)
China	1127·6 (864·4–1465·7)	14·7% (2·7–34·6)	31·5% (14·7–50·7)	0·2% (0·0–0·8)	0·4% (0·2–1·0)	1114·7 (854·3–1453·0)	6783·3 (5198·5–8842·1)
Colombia	134·1 (93·4–186·3)	47·4% (30·5–62·0)	3·9% (0·9–11·2)	0·0% (0·0–0·1)	11·5% (1·6–41·3)	86·0 (49·9–135·9)	184·3 (106·9–291·0)
Comoros	1·8 (1·7–2·1)	21·4% (20·1–24·2)	37·6% (33·6–41·2)	82·7% (62·4–96·2)	72·1% (56·2–88·2)	0·2 (0·1–0·4)	0·2 (0·1–0·5)
Congo (Brazzaville)	24·3 (18·6–31·8)	30·3% (13·5–47·5)	31·5% (21·8–44·0)	13·8% (5·6–33·7)	25·3% (12·8–44·0)	18·7 (13·2–26·1)	0·0 (0·0–0·0)
Costa Rica	35·0 (27·1–47·3)	52·0% (26·7–65·1)	28·9% (10·8–50·2)	2·8% (1·5–6·3)	9·5% (3·2–25·4)	24·8 (20·6–29·9)	83·2 (68·9–100·2)
Côte d'Ivoire	130·7 (121·4–147·4)	52·7% (52·4–52·9)	15·3% (15·0–15·7)	85·7% (75·5–91·9)	81·6% (70·0–89·3)	17·8 (10·3–29·8)	77·2 (44·8–129·1)
Croatia	14·1 (10·2–19·5)	85·4% (82·2–87·0)	2·1% (0·3–6·9)	0·0% (0·0–0·0)	0·0% (0·0–0·0)	14·0 (10·2–19·3)	9·4 (6·8–13·0)
Cuba	143·2 (101·6–197·6)	24·7% (18·7–29·4)	22·1% (8·1–36·2)	6·2% (3·5–10·8)	8·7% (3·0–25·7)	132·7 (92·6–183·3)	104·3 (72·8–144·1)
Democratic Republic of the Congo	144·4 (122·4–190·2)	41·4% (40·8–43·3)	26·2% (24·2–29·3)	78·9% (56·0–92·5)	70·4% (45·7–87·6)	10·3 (3·4–20·3)	0·0 (0·0–0·0)
Djibouti	2·3 (2·0–2·8)	28·5% (23·1–36·3)	29·0% (24·1–34·2)	30·7% (19·0–43·1)	58·5% (39·4–81·0)	0·8 (0·5–1·1)	1·7 (1·1–2·5)
Dominica	0·4 (0·3–0·5)	25·1% (12·3–39·4)	19·8% (7·4–33·7)	10·6% (4·2–24·9)	10·5% (3·5–29·6)	0·2 (0·1–0·3)	0·4 (0·2–0·6)
Dominican Republic	68·2 (47·0–103·0)	38·6% (30·7–44·6)	23·8% (11·1–37·8)	23·3% (12·5–39·6)	22·8% (7·6–58·1)	24·5 (16·1–36·1)	120·2 (78·7–176·8)
Ecuador	20·9 (14·9–28·8)	56·1% (49·1–68·9)	32·5% (15·0–43·0)	2·5% (1·4–3·9)	7·3% (3·4–18·9)	16·7 (11·4–23·8)	76·3 (52·2–108·9)
Egypt	19·8 (12·0–32·1)	26·7% (15·7–36·6)	60·3% (47·2–67·9)	3·3% (1·2–7·7)	0·4% (0·2–0·8)	17·9 (11·0–27·1)	0·0 (0·0–0·0)
El Salvador	58·8 (46·0–76·3)	38·6% (21·5–49·7)	26·1% (20·8–33·0)	5·7% (3·1–11·8)	13·0% (7·5–19·9)	49·1 (38·5–61·9)	0·0 (0·0–0·0)
Equatorial Guinea	8·4 (3·9–16·4)	37·1% (16·7–48·4)	30·9% (20·5–43·1)	0·0% (0·0–0·0)	0·1% (0·0–0·2)	7·6 (3·8–13·1)	9·8 (4·9–17·1)
Eritrea	11·7 (11·1–13·0)	24·4% (20·7–28·4)	36·6% (32·4–40·7)	74·7% (57·1–92·3)	75·4% (60·2–89·7)	1·4 (0·9–2·0)	1·3 (0·8–1·9)
Ethiopia	291·7 (272·5–323·7)	42·0% (41·5–43·3)	26·3% (24·1–30·1)	90·8% (79·3–97·8)	79·3% (62·2–91·9)	33·6 (14·9–65·0)	117·8 (52·1–227·4)
Federated States of Micronesia	0·2 (0·2–0·4)	21·6% (5·0–39·4)	12·9% (7·7–20·7)	0·0% (0·0–0·0)	0·0% (0·0–0·0)	0·2 (0·1–0·3)	0·0 (0·0–0·0)
Fiji	0·7 (0·4–1·1)	49·7% (27·4–65·7)	7·8% (2·5–16·3)	0·0% (0·0–0·0)	1·3% (0·2–4·2)	0·6 (0·4–0·9)	0·0 (0·0–0·0)
Gabon	17·3 (9·5–32·7)	32·8% (16·0–44·0)	20·1% (16·0–23·5)	0·0% (0·0–0·1)	1·0% (0·4–2·0)	14·2 (9·1–19·8)	1·5 (1·0–2·1)
Georgia	15·4 (12·8–19·1)	34·9% (26·5–42·5)	28·2% (18·0–39·1)	34·3% (21·3–52·0)	49·3% (26·1–86·2)	8·2 (6·1–10·7)	1·6 (1·2–2·1)
Ghana	97·6 (60·3–187·3)	51·7% (41·7–60·9)	20·3% (19·5–21·5)	22·0% (8·8–39·1)	54·7% (24·8–82·6)	26·1 (7·6–55·1)	19·6 (5·7–41·5)
Grenada	0·4 (0·3–0·6)	28·7% (13·3–39·6)	23·9% (8·3–39·2)	10·8% (4·9–25·5)	11·3% (3·9–32·4)	0·3 (0·2–0·3)	0·4 (0·3–0·5)
Guatemala	54·7 (41·0–72·3)	32·7% (21·3–41·5)	23·2% (14·7–32·7)	14·9% (8·3–28·4)	34·5% (16·6–66·4)	37·9 (26·6–53·1)	24·3 (17·1–34·1)
Guinea	30·7 (27·8–36·1)	32·3% (30·7–35·0)	31·3% (30·5–32·4)	78·6% (61·4–90·4)	84·8% (69·5–95·5)	3·4 (1·7–5·9)	4·9 (2·5–8·6)
Guinea-Bissau	5·3 (4·7–6·5)	55·0% (53·1–56·5)	11·5% (9·0–15·7)	87·4% (73·4–95·7)	61·5% (35·0–85·4)	1·0 (0·4–2·0)	0·9 (0·4–1·9)
Guyana	9·8 (7·6–13·3)	21·2% (13·7–28·8)	17·9% (11·2–26·2)	33·7% (16·8–62·1)	42·0% (19·2–78·3)	4·0 (2·3–6·5)	4·1 (2·3–6·7)
Haiti	98·6 (97·0–100·9)	62·7% (62·7–62·8)	8·4% (8·3–9·0)	96·9% (94·6–98·3)	96·1% (87·9–99·5)	3·5 (2·0–5·3)	4·6 (2·7–7·0)
Honduras	25·6 (19·3–33·1)	41·3% (21·5–57·6)	15·2% (5·3–30·4)	3·5% (1·7–7·9)	12·8% (3·6–35·8)	14·9 (9·6–22·0)	0·0 (0·0–0·0)
India	486·8 (346·7–695·7)	13·0% (5·8–29·2)	40·2% (18·6–67·3)	33·1% (7·5–75·3)	7·5% (2·3–16·6)	334·4 (221·8–496·4)	371·1 (246·1–550·9)
Indonesia	126·3 (105·2–154·8)	30·0% (13·6–46·7)	26·0% (16·1–41·1)	24·6% (11·2–56·6)	37·1% (17·5–65·8)	83·8 (62·7–112·3)	213·4 (159·6–285·8)
Iran	99·5 (57·5–160·3)	22·4% (17·4–29·2)	58·9% (48·9–65·3)	3·0% (1·2–5·9)	3·4% (1·7–6·5)	94·4 (53·4–153·1)	0·0 (0·0–0·0)
Iraq	6·8 (3·8–12·0)	55·9% (46·9–60·9)	29·1% (14·0–39·6)	0·0% (0·0–0·1)	0·0% (0·0–0·0)	5·2 (3·2–8·1)	0·7 (0·4–1·1)
Jamaica	24·0 (17·9–34·7)	27·2% (26·8–28·0)	20·9% (16·0–26·8)	62·7% (40·9–82·2)	32·0% (16·1–52·6)	9·7 (5·2–16·7)	27·9 (14·9–47·9)
Jordan	1·8 (1·3–2·8)	31·8% (23·0–40·3)	18·1% (10·0–27·5)	8·0% (3·7–14·7)	15·5% (5·5–35·0)	1·2 (0·6–2·0)	4·6 (2·5–7·9)
Kazakhstan	24·7 (17·0–37·1)	20·7% (16·7–24·3)	25·0% (12·1–34·7)	15·8% (8·2–26·5)	19·3% (7·7–48·3)	21·1 (13·4–33·6)	0·0 (0·0–0·0)
Kenya	759·8 (629·8–995·3)	43·9% (43·3–45·1)	23·6% (17·9–29·0)	77·0% (56·5–93·1)	49·9% (30·1–76·1)	146·0 (82·7–233·6)	29·6 (16·8–47·4)
Kiribati	0·1 (0·1–0·1)	17·2% (5·2–30·3)	38·9% (26·9–51·5)	0·0% (0·0–0·0)	0·0% (0·0–0·0)	0·1 (0·0–0·1)	0·0 (0·0–0·0)
Kyrgyzstan	16·0 (14·6–18·0)	12·9% (9·4–18·5)	24·6% (14·9–32·7)	53·1% (31·6–77·0)	35·6% (22·5–60·9)	5·0 (3·7–6·8)	0·0 (0·0–0·0)
Laos	6·7 (6·0–7·8)	20·0% (16·6–24·6)	27·6% (27·2–27·8)	55·3% (38·0–72·8)	86·8% (73·9–95·7)	1·4 (0·7–2·4)	1·3 (0·6–2·3)
Lebanon	9·4 (5·3–15·4)	86·5% (83·3–88·0)	8·1% (3·7–16·1)	0·2% (0·1–0·3)	6·9% (1·5–19·5)	8·9 (5·1–14·0)	10·2 (5·8–16·1)
Lesotho	81·5 (66·8–100·8)	49·2% (48·7–50·2)	14·8% (10·0–21·5)	68·8% (53·1–82·5)	47·8% (24·9–79·8)	29·8 (15·1–49·1)	1·1 (0·6–1·9)
Liberia	12·2 (10·3–15·3)	18·8% (10·7–27·1)	10·7% (4·1–19·7)	17·4% (8·9–33·2)	9·6% (3·4–24·0)	1·5 (0·5–3·1)	0·3 (0·1–0·6)
Libya	3·6 (2·0–6·0)	57·7% (47·5–64·7)	31·4% (18·1–44·5)	0·6% (0·3–1·3)	4·1% (1·4–10·3)	3·4 (1·9–5·5)	3·7 (2·1–6·0)
Macedonia	3·4 (2·7–4·2)	58·3% (51·3–63·8)	9·6% (9·0–12·5)	11·1% (8·1–15·6)	83·4% (50·3–98·2)	2·3 (1·6–3·0)	0·2 (0·1–0·3)
Madagascar	5·4 (4·3–7·6)	19·7% (16·2–27·4)	36·3% (28·6–45·3)	60·1% (29·2–87·7)	49·2% (26·5–75·0)	2·0 (0·9–4·2)	0·0 (0·0–0·0)
Malawi	201·7 (183·8–229·6)	36·5% (34·8–40·5)	20·5% (15·1–26·8)	83·4% (65·6–95·4)	54·4% (35·4–78·1)	25·2 (13·6–42·3)	0·0 (0·0–0·0)
Malaysia	58·4 (45·6–72·5)	55·4% (38·6–66·4)	14·7% (9·5–22·9)	1·1% (0·7–2·0)	6·8% (3·2–12·4)	55·7 (43·1–69·6)	71·2 (55·1–89·0)
Maldives	1·5 (1·2–2·0)	35·2% (20·8–48·3)	23·1% (13·1–35·1)	34·6% (17·1–67·5)	24·9% (10·8–49·3)	0·9 (0·6–1·4)	1·1 (0·7–1·8)
Mali	33·7 (29·9–40·0)	35·3% (33·6–37·5)	14·8% (11·9–18·8)	71·7% (56·6–84·7)	43·8% (28·7–59·8)	7·9 (4·5–12·4)	0·0 (0·0–0·0)
Marshall Islands	0·2 (0·1–0·4)	31·2% (10·2–46·8)	30·3% (17·3–42·2)	0·0% (0·0–0·0)	0·0% (0·0–0·0)	0·2 (0·1–0·3)	0·0 (0·0–0·0)
Mauritania	3·5 (3·0–4·3)	41·0% (36·2–46·3)	12·2% (11·5–15·1)	51·6% (37·0–68·5)	81·5% (53·9–96·9)	1·2 (0·7–1·9)	1·6 (0·9–2·6)
Mauritius	9·3 (6·4–13·4)	43·0% (28·4–53·9)	19·5% (11·5–27·8)	5·7% (2·9–11·4)	20·6% (8·8–44·1)	8·0 (5·2–12·0)	4·3 (2·8–6·4)
Mexico	522·7 (383·5–711·0)	54·7% (50·4–58·4)	13·4% (10·9–15·4)	0·0% (0·0–0·0)	0·2% (0·1–0·3)	414·5 (287·4–594·3)	286·6 (198·7–410·9)
Moldova	8·3 (6·6–11·1)	33·9% (27·9–40·4)	17·8% (16·7–20·1)	31·3% (18·8–46·0)	89·5% (68·1–98·5)	3·9 (2·2–6·8)	4·9 (2·8–8·4)
Mongolia	2·6 (2·1–3·3)	28·3% (20·3–37·8)	40·4% (29·3–49·0)	40·0% (22·3–64·5)	46·1% (28·4–74·4)	1·1 (0·7–1·6)	0·1 (0·1–0·2)
Montenegro	1·8 (1·2–2·6)	75·0% (69·9–76·6)	2·2% (0·3–8·2)	0·0% (0·0–0·0)	0·0% (0·0–0·0)	1·8 (1·2–2·5)	0·0 (0·0–0·0)
Morocco	15·8 (13·2–19·8)	30·8% (24·5–36·7)	45·7% (39·2–49·5)	18·6% (12·1–27·2)	38·2% (27·3–52·4)	8·7 (6·6–11·5)	8·4 (6·4–11·1)
Mozambique	377·2 (367·4–395·7)	38·2% (37·9–38·7)	11·2% (10·6–12·2)	93·3% (87·8–96·4)	85·4% (74·4–92·8)	21·7 (11·8–40·5)	67·0 (36·5–124·9)
Myanmar	76·1 (73·1–80·0)	21·1% (19·0–23·9)	28·4% (27·4–30·4)	82·7% (69·3–95·3)	88·2% (78·3–95·0)	5·0 (2·5–8·7)	33·6 (16·6–58·0)
Namibia	189·2 (140·4–266·3)	43·8% (30·0–55·3)	22·7% (9·1–39·4)	40·8% (20·8–74·0)	27·4% (8·6–70·2)	122·3 (81·5–179·0)	0·0 (0·0–0·0)
Nepal	9·3 (7·1–14·6)	27·5% (13·8–43·8)	18·2% (9·1–31·6)	29·5% (9·7–64·3)	35·3% (10·3–74·7)	1·4 (0·5–3·0)	3·9 (1·3–8·2)
Nicaragua	36·6 (31·0–43·1)	19·4% (14·0–25·9)	30·7% (24·1–37·5)	16·9% (10·2–26·3)	17·9% (12·0–26·1)	19·5 (14·6–24·7)	22·2 (16·7–28·2)
Niger	10·0 (8·8–12·5)	21·5% (17·9–27·3)	30·6% (27·6–34·6)	63·3% (38·7–84·4)	71·2% (49·6–88·7)	1·6 (0·6–3·4)	1·6 (0·6–3·3)
Nigeria	360·0 (329·0–412·6)	50·2% (49·1–51·0)	17·7% (16·8–19·6)	88·9% (79·2–95·6)	83·3% (65·6–95·5)	57·4 (28·3–104·7)	232·9 (115·0–425·0)
North Korea	3·4 (2·7–4·3)	32·7% (7·9–59·6)	11·9% (4·1–27·2)	0·0% (0·0–0·0)	0·0% (0·0–0·0)	2·9 (2·6–3·3)	3·0 (2·6–3·4)
Pakistan	22·9 (17·6–34·6)	24·4% (17·2–37·4)	32·1% (25·5–41·0)	37·4% (14·3–61·8)	57·0% (27·6–87·6)	11·0 (5·7–22·8)	4·6 (2·4–9·5)
Palestine	2·9 (2·1–4·4)	52·7% (37·9–59·7)	34·7% (17·9–45·3)	0·9% (0·5–1·6)	4·3% (1·9–10·3)	2·5 (1·9–3·2)	0·0 (0·0–0·0)
Panama	48·0 (30·5–71·0)	49·0% (38·4–55·8)	27·0% (21·3–33·9)	0·2% (0·1–0·4)	0·4% (0·2–0·7)	42·3 (27·6–63·3)	44·5 (29·0–66·6)
Papua New Guinea	68·1 (62·1–76·1)	13·6% (6·4–21·7)	5·6% (4·1–9·5)	21·4% (10·7–44·7)	65·4% (32·3–92·0)	14·4 (8·6–22·5)	0·0 (0·0–0·0)
Paraguay	13·6 (8·4–21·8)	45·2% (27·8–58·6)	3·0% (1·3–6·3)	0·3% (0·1–0·7)	7·7% (1·7–21·1)	7·9 (4·3–13·1)	21·3 (11·7–35·4)
Peru	64·4 (34·7–115·0)	45·2% (24·3–57·1)	35·5% (20·0–48·7)	0·1% (0·0–0·3)	1·1% (0·4–2·9)	56·5 (30·0–107·6)	84·6 (44·9–161·0)
Philippines	10·7 (7·7–14·8)	13·8% (6·6–21·9)	54·0% (49·0–58·0)	15·6% (5·9–36·9)	4·5% (2·9–6·6)	8·8 (6·3–12·3)	39·2 (28·0–55·3)
Romania	93·9 (68·6–124·4)	82·4% (81·3–83·1)	0·6% (0·2–1·8)	0·0% (0·0–0·0)	30·7% (4·7–71·5)	93·6 (68·3–123·9)	0·0 (0·0–0·0)
Russia	495·1 (280·7–827·3)	34·6% (22·4–43·3)	23·8% (10·8–35·1)	0·3% (0·1–0·7)	0·8% (0·2–2·3)	490·9 (276·6–824·5)	726·7 (409·5–1220·4)
Rwanda	142·2 (127·7–163·1)	45·6% (45·3–46·9)	27·0% (21·8–32·3)	82·9% (70·1–91·5)	54·5% (38·9–73·5)	32·1 (17·8–53·5)	0·0 (0·0–0·0)
Saint Lucia	0·6 (0·4–0·8)	30·0% (14·0–42·0)	24·8% (9·3–38·6)	8·0% (3·4–19·9)	9·6% (3·4–27·2)	0·4 (0·2–0·5)	0·8 (0·5–1·0)
Saint Vincent and the Grenadines	0·7 (0·5–1·2)	10·1% (5·4–16·9)	8·7% (3·8–18·5)	19·3% (5·7–45·8)	21·8% (4·6–57·1)	0·6 (0·3–1·0)	0·0 (0·0–0·0)
Samoa	0·6 (0·4–0·8)	13·3% (6·8–24·0)	11·7% (2·5–28·0)	0·0% (0·0–0·0)	0·0% (0·0–0·0)	0·3 (0·2–0·5)	0·1 (0·0–0·1)
São Tomé and Principe	0·4 (0·4–0·6)	32·9% (29·4–36·6)	30·4% (28·1–33·2)	70·7% (49·2–88·7)	76·5% (54·7–93·1)	0·1 (0·1–0·2)	0·0 (0·0–0·0)
Senegal	21·4 (18·7–26·1)	22·5% (18·2–27·7)	24·6% (19·6–30·6)	62·4% (40·4–85·7)	58·5% (37·3–81·5)	5·0 (2·0–10·0)	6·9 (2·8–14·0)
Serbia	9·0 (6·5–12·4)	70·9% (69·5–71·8)	2·4% (1·3–6·1)	0·0% (0·0–0·0)	43·5% (9·2–84·2)	8·8 (6·3–12·2)	21·8 (15·7–30·3)
Sierra Leone	15·8 (15·4–16·5)	28·2% (27·6–29·0)	29·5% (29·0–30·4)	93·3% (86·8–97·6)	94·0% (87·3–97·9)	0·7 (0·4–1·4)	3·5 (1·7–6·4)
Solomon Islands	0·2 (0·2–0·3)	26·1% (7·6–44·8)	26·4% (11·4–42·7)	0·0% (0·0–0·0)	0·9% (0·3–2·5)	0·2 (0·1–0·3)	0·0 (0·0–0·0)
Somalia	0·5 (0·4–0·8)	6·8% (4·0–11·7)	43·4% (36·3–54·9)	42·2% (14·2–75·5)	50·8% (25·0–70·6)	0·1 (0·1–0·1)	0·0 (0·0–0·0)
South Africa	2187·5 (1347·8–3548·6)	58·4% (50·3–64·7)	11·4% (10·5–12·9)	18·3% (9·4–31·7)	28·7% (14·4–46·9)	1641·6 (878·5–2833·2)	1153·5 (617·3–1991·0)
South Sudan	14·9 (14·3–16·4)	51·1% (49·9–51·8)	13·9% (10·7–18·3)	91·9% (82·4–98·1)	64·0% (42·9–84·4)	0·9 (0·7–1·1)	18·1 (13·4–22·8)
Sri Lanka	7·7 (5·7–10·7)	8·4% (7·5–12·1)	46·2% (42·2–53·7)	65·3% (31·0–90·9)	40·4% (24·4–57·0)	4·2 (2·3–7·0)	15·7 (8·5–26·4)
Sudan	17·5 (15·6–20·5)	30·6% (25·5–37·2)	33·7% (31·5–36·8)	54·8% (37·8–72·7)	76·5% (59·3–91·5)	3·0 (1·4–5·4)	5·0 (2·4–9·1)
Suriname	2·5 (1·7–3·4)	50·8% (40·7–58·5)	23·1% (7·6–37·4)	0·1% (0·1–0·1)	0·7% (0·2–2·4)	2·3 (1·6–3·1)	1·2 (0·8–1·6)
Swaziland	126·7 (99·0–167·7)	39·5% (35·4–47·1)	19·3% (17·2–26·2)	68·2% (42·2–92·9)	71·2% (38·2–94·3)	55·4 (29·1–94·0)	0·0 (0·0–0·0)
Syria	1·8 (1·4–2·5)	36·7% (27·5–45·4)	41·1% (33·1–48·8)	15·8% (8·8–26·3)	42·7% (25·1–67·4)	1·1 (0·7–1·7)	2·4 (1·5–3·8)
Tajikistan	14·1 (13·1–15·3)	23·5% (20·9–27·5)	38·2% (31·6–42·6)	74·8% (57·9–89·2)	63·7% (51·7–81·9)	3·5 (2·6–4·7)	0·0 (0·0–0·0)
Tanzania	449·0 (430·8–480·6)	48·4% (48·3–48·6)	27·9% (26·3–29·8)	92·1% (86·3–95·6)	81·6% (71·5–90·0)	41·6 (27·4–59·2)	59·7 (39·3–84·9)
Thailand	373·4 (303·4–451·7)	56·7% (41·7–67·9)	9·3% (4·2–17·3)	0·1% (0·1–0·2)	0·7% (0·3–1·7)	367·7 (298·2–444·5)	0·0 (0·0–0·0)
The Gambia	4·6 (4·5–5·0)	30·5% (29·0–32·3)	31·2% (30·2–32·4)	85·8% (75·7–93·4)	90·3% (80·9–96·9)	0·5 (0·3–0·8)	0·2 (0·1–0·3)
Timor-Leste	3·0 (2·4–3·8)	24·6% (16·9–33·3)	29·1% (26·8–33·3)	45·4% (24·4–77·2)	63·3% (42·0–83·3)	1·3 (0·7–2·1)	2·0 (1·1–3·3)
Togo	24·8 (20·8–32·0)	31·2% (28·4–35·9)	30·3% (28·5–34·0)	68·7% (45·3–88·4)	74·5% (50·4–93·1)	4·5 (2·2–8·1)	0·0 (0·0–0·0)
Tonga	0·1 (0·1–0·1)	20·6% (4·0–41·7)	29·0% (14·6–46·0)	0·0% (0·0–0·0)	0·0% (0·0–0·0)	0·1 (0·0–0·1)	0·0 (0·0–0·0)
Tunisia	7·9 (6·2–10·7)	48·9% (44·7–52·2)	37·6% (33·7–40·6)	2·8% (1·9–3·9)	11·8% (8·0–16·4)	5·0 (2·9–8·0)	3·9 (2·3–6·2)
Turkey	173·8 (95·4–304·3)	32·3% (22·1–41·2)	52·1% (38·2–60·6)	0·0% (0·0–0·0)	0·0% (0·0–0·0)	172·5 (95·2–298·8)	0·0 (0·0–0·0)
Turkmenistan	9·3 (5·9–14·1)	29·9% (15·2–40·4)	38·6% (16·1–53·8)	0·5% (0·2–1·4)	0·0% (0·0–0·1)	8·0 (5·3–12·0)	0·0 (0·0–0·0)
Uganda	457·3 (413·0–528·5)	45·3% (44·5–46·8)	24·0% (21·2–28·4)	85·6% (71·5–95·9)	73·6% (53·1–91·0)	35·1 (10·7–77·2)	13·5 (4·1–29·6)
Ukraine	109·6 (84·3–146·0)	38·3% (30·7–46·2)	8·4% (3·2–16·1)	21·9% (12·9–33·8)	16·7% (5·0–43·1)	51·2 (29·9–81·9)	106·7 (62·4–170·6)
Uzbekistan	20·4 (17·0–26·1)	18·7% (17·0–23·1)	48·8% (38·8–55·6)	69·6% (42·7–89·6)	50·8% (33·9–74·4)	8·6 (5·4–14·4)	4·7 (2·9–7·8)
Vanuatu	0·1 (0·1–0·2)	27·2% (7·3–44·0)	13·1% (4·3–26·7)	0·0% (0·0–0·0)	0·0% (0·0–0·0)	0·1 (0·0–0·1)	0·0 (0·0–0·0)
Venezuela	273·8 (174·8–409·1)	40·2% (24·8–57·7)	1·6% (0·2–5·6)	0·0% (0·0–0·0)	0·0% (0·0–0·0)	255·6 (161·3–386·8)	0·0 (0·0–0·0)
Vietnam	106·0 (86·4–137·2)	42·1% (37·6–46·3)	29·9% (23·1–37·8)	65·2% (44·7–87·6)	36·3% (21·3–55·1)	23·4 (11·7–40·6)	154·9 (77·5–268·6)
Yemen	2·5 (1·3–4·5)	49·9% (38·2–59·3)	35·2% (26·3–43·9)	4·2% (1·7–8·9)	17·7% (6·6–37·6)	0·7 (0·4–0·9)	0·0 (0·0–0·0)
Zambia	267·5 (250·7–296·7)	51·6% (50·9–52·5)	18·2% (15·4–22·2)	90·3% (80·5–97·4)	75·3% (55·0–93·4)	30·6 (14·1–59·2)	105·3 (48·6–203·9)
Zimbabwe	260·2 (234·2–303·2)	33·8% (31·2–37·4)	28·0% (26·7–31·1)	78·5% (60·1–93·2)	84·2% (64·7–97·1)	43·3 (23·1–72·8)	78·2 (41·8–131·7)

Data are mean (95% uncertainty interval). All spending estimates are in 2018 US$. Income groups are 2017 World Bank income groups, and Global Burden of Disease super-regions correspond to Global Burden of Disease 2017. Argentina is not captured in the GBD super-regions listed but is considered an upper-middle-income country by the World Bank and is thus captured in that grouping. $4·1 billion in development assistance was spent in administrative costs in support of HIV/AIDS programmes at the global and regional level and could not be allocated to a single country. Other spending is spending on HIV/AIDS programming that is not strictly focused on care and treatment or prevention, such as spending on health system strengthening. We excluded high-income countries, as defined by the World Bank.

**Figure 3:**
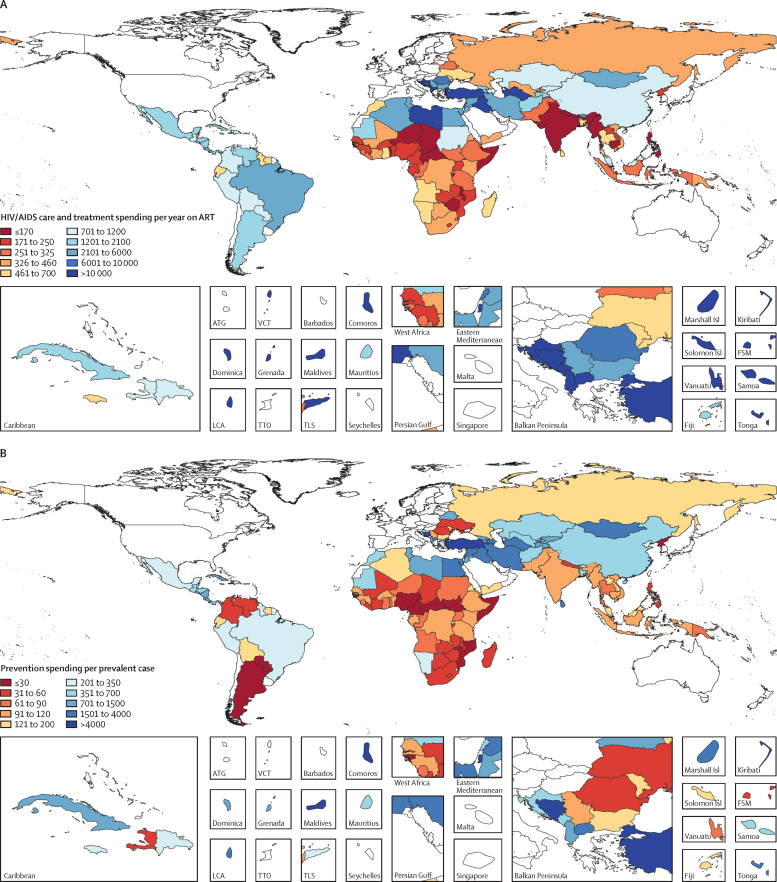
HIV/AIDS care and treatment spending per year on ART (A) and prevention spending per prevalent HIV/AIDS case (B), 2016 All spending estimates are in 2018 US$. All high-income countries, as designated by the World Bank and Global Burden of Disease, were excluded (coloured in white). ART=antiretroviral therapy. ATG=Antigua and Barbuda. VCT=Saint Vincent and the Grenadines. Isl=Islands. FSM=Federated States of Micronesia. LCA=Saint Lucia. TTO=Trinidad and Tobago. TLS=Timor-Leste.

LMICs with the lowest care and treatment spending per year on ART were most commonly located in sub-Saharan Africa, followed by south Asia and southeast Asia (figure 3A). Sub-Saharan Africa accounted for most patients on ART in LMICs in 2016 (79·2%, or 11·4 million lives). The median care and treatment spending per year on ART was $672 (IQR 284–2915). Spending varied by income and prevalence group. Low-income countries had the lowest spending on care and treatment per year on ART ($246), with countries like India and Zimbabwe spending less than $100; by contrast, middle-income countries had the highest ($2047). Spending on care and treatment per year on ART was lowest in the countries with extremely high HIV prevalence (>5% HIV prevalence), at $247 per year on ART. Spending per year on ART was $273 in high-prevalence countries (1–5% HIV prevalence) and $1244 in low-prevalence countries (<1% HIV prevalence).

Between 2000 and 2016, spending on HIV/AIDS prevention increased from $596 million (95% UI 258 million to 1·3 billion) to $3·0 billion (1·5 to 5·8 billion), a 519·6% (461·8–599·4) increase. However, as a share of total HIV/AIDS spending, spending on prevention declined from 18·5% (13·3–26·2) to 17·4% (12·2–23·72). In 2016, the median expenditure on prevention per prevalent case was $168 (IQR 66–724). Some middle-income countries spent more than $2000 on prevention per prevalent case, whereas other middle-income countries spent less than $40 on prevention per prevalent case (Argentina $21; South Africa $38; figure 3B). In sub-Saharan Africa, spending per prevalent case of HIV/AIDS varied substantially: some countries such as Mozambique, Nigeria, and Somalia spent less than $25 on prevention per prevalent case, whereas others such as Ethiopia and Namibia spent more than $110.

We estimated that, in 2016, an additional $12·1 billion (8·4–17·5) globally could be mobilised by governments of LMICs to fight HIV/AIDS. This estimate is conditional on contextual factors, such as the total government budget and total government health budget, which our analysis assumes are fixed. The ability to mobilise these potential resources was concentrated in ten middle-income countries (Argentina, China, Colombia, India, Indonesia, Mexico, Nigeria, Russia, South Africa, and Vietnam). The southeast Asia, east Asia, and Oceania region accounted for 61·2% (55·5–66·6) of these potential resources. The potential for additional government resources varied by income group: 125·0% (118·9–134·1%) for upper-middle income, 129·9% (125·5–135·8%) for lower-middle income, and 127·4% (125·4–132·4%) for low income. Across all low-income and middle-income countries, about 75% of the additional potential government spending ($10·2 billion; 7·2–14·2) could be mobilised from middle-income countries, whereas less than 5% ($390·5 million; 205·2–685·9) could be mobilised from low-income countries. Further, extremely high-prevalence (>5%), low-income countries could increase their contributions to HIV/AIDS by $145 million (78–257).

**Figure 4:**
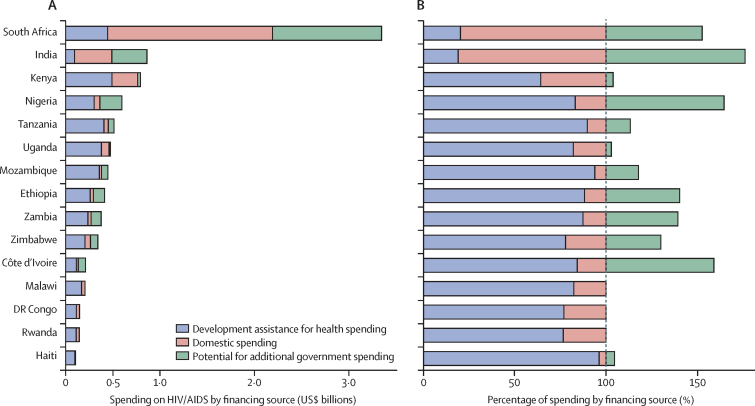
Potential government spending on HIV/AIDS relative to existing development assistance and government HIV/AIDS spending, 2016 Data are from the 15 countries that received the most development assistance for health for HIV/AIDS in 2016. Observed HIV/AIDS care and treatment spending by development assistance and domestic spending compared with the potential for additional government spending (A), and as a percentage of observed total spending on HIV/AIDS care and treatment (B). We modelled the potential for additional government spending on HIV/AIDS relative to the current government health budget, public finance, health system, and HIV/AIDS contextual factors. All spending estimates are in 2018 US$.

We estimated that governmental spending on HIV/AIDS could be increased by 111·0% in India and 405·8% in Nigeria, which are both middle-income countries (figure 4). By contrast, the governments of Uganda, Kenya, Malawi, Rwanda, and the Democratic Republic of the Congo have little capacity to fill a substantial financial void left by development assistance.

## Discussion

Across all LMICs, $19·9 billion (15·8–26·3) was spent on HIV/AIDS in 2016, of which $3·0 billion (1·5–5·8) was spent on prevention and $7·2 billion (4·3–11·8) on care and treatment. Substantial variation characterises prevention spending per prevalent case and spending on care and treatment per year on ART: high-prevalence countries like Mozambique and South Africa spent less than $40 on prevention spending per prevalent case of HIV/AIDS, whereas Ethiopia spent more than $110 on prevention per prevalent case. Low-income countries, such as India and Zimbabwe, spent less than $100 on care and treatment per year on ART in 2016, whereas many middle-income countries spent more than $2000 on care and treatment spending per year on ART. We estimated that governments of LMICs had the capacity to spend an additional $12·1 billion (8·4–17·5), a 125·7% (121·3–133·0) increase, on the fight against HIV/AIDS (including for the care and treatment of people living with HIV/AIDS), assuming that their existing government health budgets are fixed. The potential to generate additional resources was concentrated in some middle-income countries. Governments of low-income countries have the potential to generate just $390·5 million (205·2–685·9) in additional financial resources for HIV/AIDS.

Looming over the ambitious goal to end AIDS by 2030 is the threat of continued declines in development assistance for HIV/AIDS. Development assistance for HIV/AIDS dropped by about $2·2 billion from its peak of $11·2 billion in 2012.^8^ In 2016, about 17 million people living with HIV/AIDS, almost 50% of all people living with HIV/AIDS, lived in countries where development assistance financed over 75% of spending on care and treatment. Development assistance for HIV/AIDS is primarily financed by two institutions, the United States President's Emergency Plan for AIDS Relief (PEPFAR) and the Global Fund to Fight AIDS, Tuberculosis, and Malaria, which together provided 62·7% of development assistance for HIV/AIDS prevention and 94·5% of development assistance for HIV/AIDS care and treatment in 2016.^8^ Although the budget for PEPFAR has been maintained for now, the risk of reduced US PEPFAR support would have major implications for both PEPFAR, the Global Fund, and the countries they invest in.^17^ The high level of dependence on development assistance in low-income, high-prevalence countries leaves millions of people living with HIV/AIDS vulnerable to policy decisions made by one international organisation and one country.

Although we estimated that an additional $12·1 billion (8·4–17·5) in government resources could be mobilised to fight HIV/AIDS, over 80% of these potential funds are located in ten middle-income countries (Argentina, China, Colombia, India, Indonesia, Mexico, Nigeria, Russia, South Africa, and Vietnam). Many middle-income countries (such as Botswana, Namibia, and Thailand) and low-income countries (such as Haiti, Kenya, Malawi, and Uganda) would be unable to replace even 10% of the funds development assistance contributes towards care and treatment. This result suggests that these governments do not have the capacity to fill the funding gap if development assistance declines, potentially leaving about 5 million ART patients to either self-finance care or discontinue treatment. These results highlight the concerns related to development assistance for health sustainability and the value in ongoing support.

Not all countries are vulnerable to decreased funding. We estimate that, by mobilising their potential resources, 85 LMICs could generate enough funds to finance total current spending on care and treatment. Of these 85 countries, 54 could self-finance their entire current HIV/AIDS response; however, these countries are nearly all low-burden, middle-income countries. Our analysis highlights that, although development assistance finances a substantial portion of HIV/AIDS programmes in many countries, the threat posed by declines is heterogeneous. Many governments of middle-income, low-burden countries could mobilise enough domestic resources to mitigate future declines in development assistance.

In addition to preserving current levels of treatment, governments could use their available spending capacity to make progress toward the goal of ending AIDS by 2030. UNAIDS estimated that, in 2016, $23 billion would be required to finance the fast-track approach, a plan that outlines a financial path to rapidly scaling up treatment and prevention services in LMICs by 2020.^3^, ^4^ We estimate that, by governments realising their full spending potential, 38 countries could have achieved country-specific funding goals with current or reduced development assistance in 2016.

Redirection of funding away from countries capable of achieving UNAIDS funding targets could release about $1 billion in development assistance in 2016. However, focusing on country-specific funding only, these funds are not enough to cover the nearly $5·5 billion required to fund the UNAIDS fast-track approach in 78 countries that are unable to achieve the funding targets and are home to nearly 70% of people living with HIV/AIDS. To bridge this financing gap and make progress toward ending AIDS by 2030, the only immediate source of financing available is more development assistance. Indeed, a recent analysis showed that donors have the potential to contribute an additional $13·3 billion.^18^ In the process of allocating funds, our estimates might help the donor community to identify the countries that are most in need of international investments and those countries that are ready to transition away from development assistance.

Governments with the potential to spend additional HIV/AIDS resources could raise these resources in various ways. First, governments might consider instituting new taxes earmarked for HIV/AIDS, including taxes on alcohol or cigarettes, or improving the collection of taxes through changes to collection systems and investment in enforcement. Reallocating funds to HIV/AIDS from the broader health and government budget are also potential strategies, albeit they involve challenging tradeoffs with potential for adverse consquences.^15^, ^19^, ^20^ Finally, given that the income of all LMICs is expected to grow by 2030, this additional income could be used by governments to raise more revenue and provide more funding for HIV/AIDS programmes.

The generation of additional government resources will not occur without deliberate and strategic planning. Many governments face serious challenges for raising-revenue that require more fundamental reforms to governance and face competing external pressures from donors to increase domestic spending on a range of health and development issues. HIV spending decisions do not, nor should they, occur in a vacuum. Policy makers in countries where we estimate additional resources could be mobilised should carefully consider the available options for raising additional revenue, their viability, and the associated tradeoffs.

Our estimates of governments' potential spending on HIV/AIDS are not intended to reflect what governments should spend on HIV/AIDS. Instead, our analysis aims to measure what governments could theoretically spend on HIV/AIDS, conditioned on countries' HIV/AIDS burden and contextual factors, such as budgetary pressures and financial capacity of health systems, governments, and economies. By conditioning our analysis on various factors, we offer a more conservative estimate of the potential for governments to spend additional resources on HIV/AIDS compared with analysis based exclusively on income and HIV/AIDS indicators. Furthermore, what these factors also suggest is that, over time as incomes rise and countries prioritise health spending, an increasing amount of funds will be available for HIV/AIDS. These funds will help countries progress towards reaching the UNAIDS goals of ending AIDS by 2030. By making cross-country comparisons, these estimates are intended to serve as an initial step in framing the discussion around appropriate levels of future government spending on HIV/AIDS.

The provision of additional funds to fight HIV/AIDS alone will not end the AIDS epidemic. Health systems receiving additional funds must have the capacity to absorb the inflow of resources to ensure funding is spent effectively and efficiently. As an analysis^21^ showed, substantial inefficiencies affect the delivery of ART services in three low-income and high HIV/AIDS burden countries and could limit the number of patients receiving ART by nearly 30%. Further research might expose new approaches to realising efficiency gains, boosting the impact of investments in HIV/AIDS, and increasing global capacity to meet global HIV/AIDS targets. Expansion of ART coverage is not the only important aim. Substantial investment should be made to improve the quality of ART care to achieve the UNAIDS target of 90% viral suppression, which is crucial to reducing the transmission of HIV/AIDS and ending the epidemic. Overall, efficient translation of financing into effective HIV/AIDS prevention and treatment will be key to maximising the finite financial resources available.

Our estimate of total HIV/AIDS spending in LMICs is lower than our previous estimate ($17·4 compared with $19·1 billion in 2015, measured in 2015 US$) and the UNAIDS estimate ($19·7 billion in 2015, measured in 2015 US$).^22^ Given that the underlying data estimation methods have not changed, the differences are probably attributable to the 3204 additional datapoints that were added to our models. Despite the reduction in our global estimates of HIV/AIDS spending, the country-level median change between the estimates of HIV/AIDS spending is about 6%. This disparity is despite our decision to attribute administrative costs of HIV/AIDS occurring outside the borders of a country to global HIV/AIDS spending rather than country-level HIV/AIDS spending, a step taken to better align estimates of spending with System of Health Accounts definition of health spending.

Differences in study objectives and study designs make direct comparisons between our estimates of potential spending and the result of previous studies^15^, ^23^, ^24^, ^25^ difficult. Our study contrasts with previous studies in seeking to estimate how much countries could spend, not how much countries should spend, nor how spending compares with global benchmarks.^23^, ^25^ Further, what is being measured varies among studies, because some analyses estimate the potential for additional domestic resources, whereas others estimate the potential for additional government resources.^15^, ^23^, ^24^, ^25^ In our analysis, we aimed to address the latter question because the former question implicitly calls for increased out-of-pocket expenditure on HIV/AIDS. Finally, the methods used differ. Many studies^15^, ^23^, ^24^, ^25^ compare HIV/AIDS spending as a fraction of GDP to established benchmarks by regressing HIV/AIDS spending on variables such as income, prevalence, and government effectiveness. Many of these regression approaches assume that the potential for countries to spend more is completely explained by the residuals of the models and, by doing so, ignore data uncertainty—an assumption that is likely not met given that previous studies^24^, ^25^ use robust regressions, presumably to minimise the effect of data outliers.

To estimate what governments could spend on HIV/AIDS, we implemented stochastic frontier analysis, a statistical modeling approach that captures both data uncertainty and models the potential for governments to spend more resource on HIV/AIDS. Any claims about the potential for governments to spend more on HIV/AIDS should be qualified by the limitations of stochastic frontier analysis. The validity of this analysis is dependent on identifying the correct model specification. Although we guarded against model misspecification by testing multiple specifications and chose the model based on objective criteria, there is no way of knowing the exact model specification. Further, stochastic frontier analysis requires an assumption about the distribution of efficiency estimates used to calculate the potential for additional HIV/AIDS spending. There is no way of knowing the distribution of these efficiency scores, but we chose the most flexible distribution to fit an array of potential distributions.

Despite the difference in our approach to estimating potential spending on HIV/AIDS, our estimates are, on average, 50% lower than previous estimates (appendix). This result is surprising and alarming because our analysis theoretically suggests a ceiling of available resources, or maximum potential. The fact that our estimates of the potential HIV/AIDS spending are lower than estimates that take a more moderate approach is worrisome because it indicates that fewer resources are available than previously thought.

Comparison of our potential spending estimates with UNAIDS estimates of the required resources to reach global HIV/AIDS goals is by no means perfect.^3^ Neglecting the limitations of our analysis, UNAIDS note that “significant uncertainty” not captured in their analysis surrounds their estimates of the required resources to reach global HIV/AIDS goals. Uncertainty that is primarily driven by how well countries scale up HIV/AIDS services, which could lead to cost overruns of scale-up efforts and place the achievement of HIV/AIDS goals further out of reach.

Finally, our estimates of HIV/AIDS spending and estimates of potential for government spending, are based on imperfect data. For example, the reported data might not fully capture spending, over report spending from relevant financing source, or not appropriately classify spending into appropriate spending categories. In our analysis, we sought to address these data deficiencies by producing estimates using all available data so that no one datapoint would overly influence our estimates. Throughout the analysis, we quantified, propagated, and presented uncertainty in a transparent manner. Although this approach might have led to large UIs, it is indicative of the state of the quality and availability of data in global health and serves as a reminder of the need for comparable and rigorous data collection systems.

Between 2005 and 2016, development assistance cumulatively financed $108·1 billion of the $193·7 billion (160·8–244·6) spent on HIV/AIDS in LMICs.^11^ This unprecedented commitment by the international community helped to fuel large declines in HIV/AIDS mortality and incidence. Although LMICs have made substantial contributions to the fight against HIV/AIDS, many are unable to finish this fight alone. Sustained contributions by the international community and by governments of LMICs will be required to end AIDS by 2030.
