# Supplementary material for: Potential for additional government spending on HIV/AIDS in 137 low-income and middle-income countries: an economic modelling study
Source: Lancet HIV. 2019 Apr 26;6(6):e382–95. doi: 10.1016/S2352-3018(19)30038-4 (PMC6540601; doi:10.1016/S2352-3018(19)30038-4)
Supplement: Supplementary appendix [file mmc1.pdf]

THE LANCET
HIV

Supplementary appendix

This appendix formed part of the original submission and has been peer reviewed. We post it as supplied by the authors.

Supplement to: Haakenstad A, Moses MW, Tao T, et al. Potential for additional government spending on HIV/AIDS in 137 low-income and middle-income countries: an economic modelling study. *Lancet HIV* 2019; published online April 25. http://dx.doi.org/10.1016/S2352-3018(19)30038-4.

Supplementary material for

Potential for additional government spending on HIV/AIDS in 137 low-income and middle-income countries: an economic modelling study

Table of Contents

Acronyms and abbreviations 2
Overview of data sources and data cleaning process 3
Currency conversion 6
Modeling HIV/AIDS spending with ST-GPR 6
Enforcing internal consistency 8
Development assistance for health 10
Estimating potential government spending 11
Stochastic frontier analysis model selection 11
Comparison of potential spending to the literature 12
Results in Purchasing price parity 13
Work cited 17

Acronyms and abbreviations

BIC, Bayesian information criteria
DAH-HIV/AIDS, Development assistance for health for HIV/AIDS
GAM, Global AIDS monitoring
HAQI, Healthcare access and quality index
HIV/AIDS, Human immunodeficiency virus, acquired immunodeficiency syndrome
LCU, Local currency units
LDI, 10-year lag distributed GDP per capita
NASAs, National AIDS Spending Assessments
NHA, National Health Accounts
PPP, Purchasing price parity
SFA, Stochastic frontier analysis
ST-GPR, Spatiotemporal Gaussian Process Regression
THE, Total health expenditure
UNAIDS, the Joint United Nations Programme on HIV/AIDS
USD, United states dollar

Overview of data sources and data cleaning process

In this study, we were interested in extracting data providing information on HIV/AIDS spending at the country-level by domestic financing source (domestic, government, private, prepaid private and out-of-pocket (OOP)) and domestic spending by spending category (care and treatment, prevention, and other). We extracted publicly available data from websites of international institutions and government data aggregators. HIV/AIDS spending data were extracted from seven sources:

- AIDSinfo database published by the Joint United Nations Programme on HIV/AIDS (UNAIDS)
- UNAIDS health financing dashboard
- Global AIDS monitoring (GAM)
- Government and private spending data reported by countries in proposals and concept notes submitted to the Global Fund to Fight AIDS, Tuberculosis and Malaria (the Global Fund)
- National Health Accounts that capture HIV/AIDS spending, including sub-accounts and accounts that adhere to the System of Health Accounts 2011 (SHA 2011) methodology
- National AIDS Spending Assessments (NASAs)
- Asia Pacific region data downloaded from the AIDS data hub

We leveraged the unique strengths across the different datasets, with the understanding that they were all generated to serve different purposes. The financing data collated by UNAIDS is sourced from annual reporting by countries to UNAIDS, in line with the 2000 Declaration on Commitment to HIV/AIDS. Similarly, countries report domestic spending in concept notes and proposals submitted to the Global Fund to secure funding. The Global Fund requires countries submit these estimates as part of a requirement that they contribute funds to the disease area of focus, in addition to Global Fund contributions. Staff at both the Global Fund and UNAIDS verify the data submitted to them, but in general do not publish data that has been altered from what countries themselves report.

Not all extracted data sources used the same definition of health expenditures. For example, National AIDS Spending Assessment's (NASAs) definition of health expenditure on HIV/AIDS followed a broader definition than the definition of health expenditure provided by National Health Accounts (NHAs). Specifically, NASAs included expenditure on non-health spending categories such as orphans and vulnerable children, creation of an enabling environment, and other social protection services.

To harmonize the definition of HIV/AIDS related health expenditure amongst data sources, when provided, we subtracted expenditure related to orphans and vulnerable children, creation of an enabling environment, and social protection services, from the respective sources and functions of health expenditure reported in the NASAs. The three spending categories of orphans

and vulnerable children, creation of an enabling environment, and social protection do not represent an exhaustive list of the deviations between NASAs and NHAs' HIV/AIDS spending definition, but do represent the vast majority of this deviation. Other spending categories that were included in NASAs but not included in NHAs were more granular and frequently not reported. Finally, in inspecting some of our data, we noticed the presence of duplicates. This was because many of our data sources drew from the same underlying data (AIDSinfo, UNAIDs' health financing dashboard, NASAs, GAM). We guarded against the inclusion of duplicates by excluding data points that overlapped perfectly or when the value for the previous year was copied for the subsequent year.

We extracted a total of 8,589 unique data points. Data for government health spending on HIV/AIDS was most substantial, with more than 3,500 data points. The fewest data points existed for HIV/AIDS care and treatment and prevention, respectively amounting to 1,016 and 863 data points. eTable 1 provides breakdowns of the number of data points by year and quantity of interest.

eTable 1 captures the availability of HIV/AIDS spending data by country. Data density was highest in low- and middle-income countries with a large HIV/AIDS burden, notably Southern and Eastern sub-Saharan Africa; high-income countries, with the exception of the United States, had the fewest data points.

Year	Domestic spending	Government spending	Private spending	Out-of-pocket spending	Prepaid private spending	Domestic care and treatment spending	Domestic prevention spending	Domestic other spending
1996	1	1	1	1	1	0	0	0
1997	1	1	1	1	1	1	1	0
1998	1	1	1	1	1	1	1	0
1999	2	6	2	2	1	1	1	0
2000	7	13	5	4	1	7	7	1
2001	24	13	6	2	2	9	9	2
2002	25	16	10	7	2	12	12	5
2003	27	18	8	2	2	7	9	3
2004	50	49	13	3	4	9	13	4
2005	61	125	42	6	10	36	29	10
2006	65	262	77	13	14	65	56	17
2007	56	341	100	15	20	102	71	16
2008	71	424	165	20	23	154	140	33
2009	76	420	175	18	19	153	129	28
2010	86	377	180	18	19	141	120	33
2011	67	322	154	12	16	116	96	23
2012	115	329	194	25	27	99	79	27
2013	109	286	136	14	15	66	50	13
2014	111	225	105	10	15	25	27	11
2015	93	132	65	6	6	12	13	7
2016	35	81	31	0	0	0	0	0
2017	4	72	26	0	0	0	0	0

eFigure 1: Map of HIV/AIDS data availability

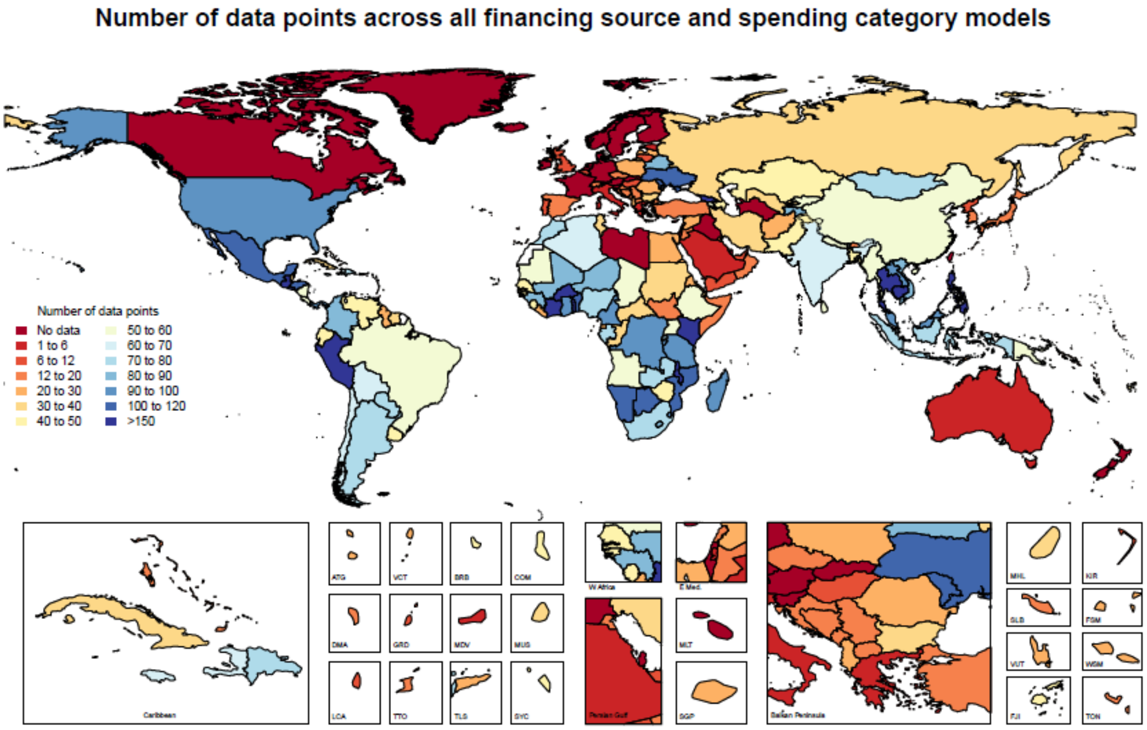

While the analysis presents on only domestic and government spending on HIV/AIDS in 137 low- and middle-income countries, we used data from high-income countries to inform and improve the overall fit of the models.

Currency conversion

All HIV/AIDS expenditure estimates are presented in 2018 United States dollars. Data sources reported spending in either nominal local currency units (LCUs) or nominal United States Dollars (USD). To convert nominal LCUs to United States dollars, we applied deflators to nominal LCU to inflate to 2018 LCUs. We then applied exchange rates to produce 2018 United States dollars. When LCUs were not reported, we extracted reported expenditure in nominal USD, applied corresponding nominal exchange rates to produce nominal LCUs, inflated nominal LCUs to 2018 LCUs with deflators, and finally exchanged 2018 LCUs to 2018 United States dollars. All deflators and exchange rates were extracted from the World Bank[1], International Monetary Fund[2], Penn World Tables,[3] the United Nations National Accounts,[4] and the World Health Organization,[5] and were imputed to provide a complete series for each of the variables between 1950 and 2018. We then used several models including ordinary least-squares regression and mixed effects models, to complete each source series from 1950 to 2018. More information about these converters and deflators may be found here[6]

Modeling HIV/AIDS spending with ST-GPR

As previously mentioned in the manuscript, we used spatiotemporal Gaussian process regression (ST-GPR) to model HIV/AIDS spending for five financing source models (domestic, private,

government, prepaid private, and OOP) and three domestic spending category models (prevention, care and treatment, and other). The interested reader may view a complete description of ST-GPR here[7]. Briefly, ST-GPR has three primary steps. First, a linear mixed effects model is run with a given set of predictors. Predictions from the first step provide the general trend within the data. In the second step, spatiotemporal patterns were estimated by applying a series of spatiotemporal weights to average the residuals of the first step linear model. These spatiotemporal patterns were then added to the linear prediction to generate spatiotemproal predictions. Finally, the spatiotemporal predictions served as the mean function of a Gaussian process regressions run across time on the data. Estimates of the Gaussian process regressions served as final ST-GPR predictions and generated a complete time-series of data from 2000 to 2016 in 137 countries, building from data when available and borrowing strength across time, geographic regions, and covariates' predictive power when data was not available.

For the first step of ST-GPR—the linear model—we used a linear mixed effects regression with random effects on Global burden of disease (GBD) super region and GBD region. For prediction in these models, we considered a range of covariates: ten-year lagged distributed income (LDI), HIV prevalence, incidence, mortality, and antiretroviral coverage and price. The exact set of covariates for each model was determined based on out-of-sample predictions and presented in eTable 2. All covariates were sourced from GBD 2017[8,9]. Within the linear model step, we measured each data point's cook's distance, and after checking to ensure the data was properly extracted, we excluded every data point with a cook's distance exceeding 4/n, where n is the total number of data points within the model.

In the second step, we created spatiotemporal predictions by smoothing the predictions from the first step model based upon systematic deviations in the residuals of the first step model across time and geographic locations. The spatiotemporal predictions were passed as the mean function to a Gaussian process regression along with the data to produce final ST-GPR predictions. For every country-year estimate, 1,000 draws were generated from the Gaussian process regression model, which were later used in subsequent calculations.

eTable 2: Covariates used in ST-GPR

ST-GPR model	Covariates
Domestic	ART price, ART coverage, HIV/AIDS prevalence, HIV/AIDS mortality rate
Government	ART coverage, HIV incidence, HIV/AIDS mortality
Private	LDI, ART price, HIV/AIDS prevalence
OOP	ART coverage, HIV/AIDS prevalence, HIV incidence, HIV/AIDS mortality rate
Prepaid private	ART price, ART coverage, HIV/AIDS prevalence, HIV incidence, HIV/AIDS mortality
Domestic HIV/AIDS spending on care and treatment	ART coverage, HIV/AIDS prevalence, HIV incidence, HIV/AIDS mortality rate
Domestic HIV/AIDS spending on prevention	ART coverage, HIV/AIDS prevalence, HIV incidence, HIV/AIDS mortality rate
Domestic HIV/AIDS spending on all other functions	ART price, ART coverage, HIV incidence

Enforcing internal consistency

To ensure internal consistency between the HIV/AIDS spending estimates and the all health spending estimates[6], HIV/AIDS spending by source was modeled as the logit transformed fraction of the respective, loess smoothed, all health spending by financing source estimate (e.g. domestic HIV/AIDS spending divided by all domestic health spending). As a consistency check, extracted data points were outliered if the fraction of HIV/AIDS spending by source and all health spending by source exceeded one.

While the above transformation helped ensure internal consistency between HIV/AIDS spending and all health spending, we were still required to ensure internal consistency within our estimates such that government HIV/AIDS spending did not exceed domestic HIV/AIDS spending, and further we wished to take advantage of all the extracted data within the implemented models. These objectives were accomplished by both aggregating and raking. Aggregating is the process of summing mutually exclusive and collectively exhaustive estimates of sub-components of health expenditure (e.g.OOP, prepaid private, government spending, development assistance) and using the sum as the estimate of total health expenditure. Raking is the exact opposite of aggregating. In raking, we used estimates of total health expenditure to evenly scale the estimated sub-components to ensure the sub-components sum to the estimated total health

expenditure. Raking and aggregating are equally valid and widely used in health financing and in the Global Burden of Disease[8,9].

In our extracted dataset, few data sources (NHAs and NASAs) reported OOP, prepaid private, or total HIV/AIDS spending (sum of OOP, prepaid private, government, DAH-HIV/AIDS), while nearly all data sources reported expenditure of either government, private (sum of OOP and prepaid private, but not disaggregated), and total domestic (sum of government and private, but not disaggregated) HIV/AIDS spending. Given this inconsistency, we modeled the five financing source spending variables and raked and aggregated estimates to draw strength across areas with the highest data density. This process was implemented by averaging the domestic HIV/AIDS spending estimates with the aggregated domestic HIV/AIDS spending estimate formed by summing estimates of government and private HIV/AIDS spending. This averaged result represented our final estimate of domestic HIV/AIDS spending. We then raked estimates of government and private HIV/AIDS spending to the final domestic HIV/AIDS spending envelope to produced final private and government HIV/AIDS spending estimates. The final private HIV/AIDS spending estimates were then used as an envelope to rake OOP and prepaid private HIV/AIDS spending estimates. To propagate uncertainty, we conducted both aggregating and raking on the draw level. For estimates of total HIV/AIDS spending, we deterministically added development assistance, source from published literature[6], to total domestic HIV/AIDS spending

To generate estimate of domestic HIV/AIDS spending by spending category (prevention, care and treatment, and other), we estimated domestic spending by spending category as the logit transformed fraction of total domestic spending. Not only did these models draw on reported domestic spending by spending category, but they drew on data reporting total spending by spending category. We equated these latter data to domestic spending by spending category by subtracting estimates of development assistance from analogous spending category. This process required us to map development assistance by HIV/AIDS health focus area to our spending categories. This mapping is presented in eTable 4. If this process of equating data resulted a negative value, after checking the data point of incorrect extraction, we dropped the data point.

Estimates of domestic spending by spend category were raked to the total domestic spending envelope. Final estimates of HIV/AIDS spending by spending category were created by deterministically adding domestic spending to development assistance by spending category.

eTable 3: Aggregation of development assistance for HIV/AIDS by health focus area into HIV/AIDS spending categories.

HIV/AIDS spending functions	Development assistance for HIV/AIDS by health focus area.
Prevention	Prevention, PMTCT
Care and treatment	Treatment, Care, Counseling and Testing
Other	Health system strengthening
	Unidentified

Development assistance for health

Development assistance for health (DAH) estimates were obtained from the Institute for Health Metrics and Evaluation's development assistance for health database. A more detailed description of the methodology used to obtain the estimates in the database can be found in Dieleman et al.[6] All known, systematically reported, available data on health-related disbursements and expenditures were extracted, as well as income and revenue from existing project databases, annual reports, and audited financial statements. DAH for bilateral agencies included all health-related disbursements from bilateral donor agencies, excluding funds that they transferred to any of the other channels we tracked in order to avoid double-counting. This information was extracted from the Creditor Reporting System (CRS) and Development Assistance Committee (DAC) databases of the Development Assistance Committee of the Organisation for Economic Co-operation and Development (OECDDAC).

In some cases, donor agencies did not report disbursement data to the CRS. A method for predicting disbursements from commitment data was implemented to address this challenge. For other grant- and loan-making institutions, annual disbursements on health grants and loans were similarly included, excluding transfers to any other channels and ignoring any repayments on outstanding debts. The annual disbursements for grant- and loan-making institutions only reflect the financial transfers made by these agencies. Therefore, in-kind transfers from these institutions in the form of staff time for providing technical assistance and the costs of managing programs were estimated separately. Estimates of DAH for the United Nations (UN) agencies included annual expenditures on health both from their core budgets and from voluntary contributions. Non-governmental organizations' (NGOs) DAH estimates utilized data from US government sources, Guidestar Research Fundamental Plus dataset and a survey of health expenditure for a sample of NGOs to estimate DAH from US-based and internationally based NGOs receiving support from the US government. To allocate DAH to HIV/AIDS and the program areas within development assistance for HIV/AIDS, we used a keyword search and tagged spending based on a weighted-average of keywords. Extensive details about all the steps involved in estimating DAH are in Dieleman et al.

Estimating potential government spending

We used stochastic frontier analysis (SFA) to estimate the potential for governments to spend additional resources on HIV/AIDS. Stochastic frontier analysis (SFA) is an econometric regression method that incorporates two errors terms: (1) a normal distributed error term common in most linear regressions, and (2) a one-sided error term with an assumed truncated normal distribution. As described in the manuscript, the latter error term can then be converted into an efficiency score measured on the interval of zero to one. We used this efficiency score to estimate governments' potential to spend additional resources. We assumed the one-sided error term followed a truncated normal distribution, an assumption that is generally considered extremely flexible[10].

In our SFA regression, we regressed our log government spending on HIV/AIDS per capita against a set of covariates: LDI, HIV prevalence, HIV incidence, HIV mortality, the healthcare access and quality index[5] , general government expenditure per capita less total government spending on health per capita [8], government spending on health per capita less government spending on HIV/AIDS per capita, and dummies to denote GBD super region groups. We used the natural log of all covariates. As described in the manuscript, we additionally chose to include all possible covariate interactions in our selected model. This decision was made after testing several SFA model specifications commonly used in the literature. Specifically, we assessed the Cobb-Douglas specification (all first order terms), a quadratic specification (first and second order terms), an interaction specification (all first order terms and all possible first order interactions), and finally the translog specification (all first and second order terms and all possible first order interactions). First order terms are defined as the standard set of covariates mentioned above and second order terms are defined as the square of all first order terms after taking the natural log. These four specification were evaluated on the basis of Bayesian information criteria (BIC)—a goodness of fit statistic that penalizes complex models. As seen in eTable #4, the BIC for the interaction specification had the lowest BIC amongst all models tested, which suggested that it was the best performing model.

Stochastic frontier analysis model selection

As mentioned in the manuscript, the estimates of efficiency in stochastic frontier analyses are impacted by selection of the functional form i.e. the choice of covariates within the model. We evaluate four commonly implemented functional forms founds within the literature: Cobb-Douglas speciation (model with first order terms (maximum power is one)), quadratic specification (model with first order term and second order terms), interaction specification (model with first order terms and interactions), and a translog model (a model with first and second order terms and all possible interaction between first order terms). In comparing these models, we selected the model with the lowest Bayesian information criteria, which was the interaction model. Table S2 presents the Bayesian information criteria for each functional forms tested. Additionally, in Table S2 we estimate the additional potential government spending for all low- and middle-income countries under each model as a sensitivity analysis to display how sensitive our results are to the choice of model selection.

eTable 4: Bayesian information criteria from stochastic frontier models.

Functional form	Bayesian information criteria	Estimated potential additional government spending at the global level (measured in billions; USD 2018)
Cobb-Douglas	348	4.5
Quadratic	345	3.4
Interaction	397	4.3
Translog	313	12.1

Comparison of potential spending to the literature

As mentioned in the manuscript, comparison of our estimates of governments' potential to spend additional resources on HIV/AIDS to the existing literature proves difficult due to the methods implemented, the unit of measurements (governments vs countries total domestic spending), and the aim of the study (i.e. assessing what governments *could* spend opposed to what governments/countries *should* spend domestically). Despite these difference, in Table #S3 we compare our estimates of what governments could spend to Remme et al. 2016[11] regression based estimates of 14 governments' potential spending on HIV/AIDS. All estimates are reported in spending per person living with HIV/AIDS in 2018 USD.

eTable 5: Comparison of governments potential spending on HIV/AIDS per person living with HIV/AIDS

Country	Remme et al 2016 estimated potential spending by governments	IHME estimated potential spending by governments
Botswana	3,287	557
Ethiopia	184	230
Kenya	465	101
Lesotho	404	100
Mozambique	74	43
Malawi	105	24
Namibia	1,923	573
Nigeria	195	83
Swaziland	576	269
Tanzania	183	76
Uganda	87	37
South Africa	1223	429
Zambia	187	116
Zimbabwe	83	96

Note: All estimates are reported in 2018 USD. Remme et al 2016 estimates were retrieved using software that analyzed a figure in their analysis as estimates of governments' potential spending were not reported in a table or text.

Remme et al 2016 estimates ranged from 80% to nearly six times more than our estimates (median was 2.4 times higher). This result suggest our estimates of governments potential spending may significantly be less than previously thought.

Results in Purchasing price parity

In Table S4 we present our spending results in 2018 purchasing price parity (PPP), opposed to 2018 USD as reported in the main text.

Table of HIV/AIDS Spending in 2018 PPP dollars in 2016			
**Location**	**Total spend on HIV/AIDS in millions of USD**	**Total government spend on HIV/AIDS in millions of USD**	**Potential government spend on HIV/AIDS in millions of USD**
**Wold Bank Income Group**			
World Bank Low Income	7804.3 (7214.3 to 8801.1)	851.2 (427.8 to 1522.8)	1066.3 (566.8 to 1859.5)
World Bank Lower Middle Income	9588.0 (7842.8 to 12388.0)	3611.4 (2247.2 to 5602.4)	5083.7 (3052.9 to 8145.6)
World Bank Upper Middle Income	19793.7 (13036.5 to 30253.1)	16877.9 (10991.2 to 24957.4)	20097.4 (14110.4 to 28498.7)
**GBD Super-region**			
Central Europe, Eastern Europe, and Central Asia	2455.6 (1635.8 to 3687.2)	2005.1 (1207.0 to 3213.8)	2387.7 (1371.4 to 3950.9)
Latin America and Caribbean	7680.6 (5082.9 to 11865.3)	6095.1 (4143.7 to 8429.1)	2361.0 (1543.0 to 3528.6)
North Africa and Middle East	1217.2 (741.5 to 1968.0)	1073.9 (619.8 to 1766.8)	132.6 (79.9 to 208.3)
South Asia	2008.0 (1441.2 to 2871.7)	1337.2 (879.8 to 2010.3)	1465.2 (965.2 to 2191.9)
Southeast Asia, East Asia, and Oceania	4724.7 (3816.1 to 5881.6)	3745.2 (2872.5 to 4850.0)	14420.4 (10828.7 to 19147.8)
Sub-Saharan Africa	18451.2 (14925.9 to 24274.1)	6439.2 (3496.9 to 10923.8)	5023.9 (2625.6 to 8846.6)
**Country**			
Afghanistan	23.7 (20.1 to 31.5)	2.0 (0.8 to 3.4)	4.6 (1.9 to 7.8)
Albania	4.4 (3.0 to 6.2)	4.2 (2.9 to 5.9)	7.2 (5.0 to 10.0)
Algeria	65.8 (45.6 to 102.4)	65.2 (45.2 to 101.6)	0.0 (0.0 to 0.0)
American Samoa	0.3 (0.2 to 0.4)	0.3 (0.2 to 0.4)	0.0 (0.0 to 0.0)
Angola	162.0 (121.6 to 213.2)	112.4 (71.9 to 163.7)	0.0 (0.0 to 0.0)
Argentina	648.7 (450.2 to 894.3)	644.6 (446.5 to 889.0)	456.7 (316.3 to 629.8)
Armenia	14.5 (12.1 to 18.1)	6.7 (4.4 to 10.4)	8.1 (5.3 to 12.6)
Azerbaijan	45.5 (32.6 to 65.7)	27.7 (14.9 to 47.8)	13.5 (7.2 to 23.2)
Bangladesh	35.0 (29.3 to 42.4)	15.5 (10.3 to 22.9)	20.1 (13.4 to 29.6)
Belarus	68.0 (51.4 to 87.3)	50.0 (35.5 to 67.9)	0.0 (0.0 to 0.0)
Belize	3.8 (2.7 to 5.5)	3.0 (2.1 to 4.4)	1.7 (1.1 to 2.5)
Benin	55.4 (50.0 to 63.5)	12.2 (7.1 to 20.1)	4.6 (2.7 to 7.6)
Bhutan	5.3 (4.6 to 6.7)	2.4 (1.6 to 3.8)	0.0 (0.0 to 0.0)
Bolivia	30.4 (20.5 to 46.5)	19.3 (10.6 to 32.5)	35.6 (19.6 to 60.0)
Bosnia and Herzegovina	12.1 (8.7 to 16.9)	10.7 (7.3 to 15.6)	0.0 (0.0 to 0.0)
Botswana	596.4 (413.3 to 849.9)	458.8 (291.8 to 654.1)	0.0 (0.0 to 0.0)
Brazil	4236.5 (2583.8 to 7110.0)	3619.4 (2493.3 to 4790.5)	0.0 (0.0 to 0.0)

Bulgaria	21.5 (15.4 to 29.9)	18.5 (12.5 to 26.7)	28.8 (19.5 to 41.7)
Burkina Faso	84.8 (60.3 to 123.8)	34.1 (13.9 to 70.0)	0.0 (0.0 to 0.0)
Burundi	56.8 (53.7 to 62.3)	5.2 (2.3 to 10.6)	3.2 (1.4 to 6.5)
Cambodia	99.8 (90.3 to 113.2)	21.3 (12.5 to 32.9)	134.7 (79.4 to 208.5)
Cameroon	170.7 (148.4 to 213.6)	31.2 (13.2 to 65.9)	71.4 (30.2 to 150.7)
Cape Verde	3.0 (2.1 to 4.4)	2.8 (1.9 to 4.1)	1.5 (1.0 to 2.2)
Central African Republic	18.0 (17.4 to 19.0)	1.1 (0.7 to 1.5)	0.5 (0.3 to 0.7)
Chad	42.1 (34.6 to 56.3)	16.4 (9.2 to 30.0)	0.0 (0.0 to 0.0)
China	2088.0 (1600.7 to 2714.2)	2064.2 (1581.9 to 2690.7)	12561.2 (9626.5 to 16373.6)
Colombia	319.7 (222.7 to 444.1)	205.1 (118.9 to 323.9)	439.2 (254.7 to 693.6)
Comoros	3.6 (3.3 to 4.2)	0.4 (0.1 to 0.8)	0.5 (0.1 to 1.1)
Congo	72.1 (55.4 to 94.7)	55.7 (39.3 to 77.5)	0.0 (0.0 to 0.0)
Costa Rica	52.3 (40.5 to 70.6)	37.1 (30.7 to 44.7)	124.3 (103.0 to 149.7)
Cote d'Ivoire	302.3 (280.9 to 341.0)	41.3 (23.9 to 69.0)	178.7 (103.6 to 298.8)
Croatia	25.6 (18.6 to 35.4)	25.5 (18.5 to 35.2)	17.1 (12.5 to 23.7)
Cuba	313.6 (222.4 to 432.7)	290.5 (202.7 to 401.3)	228.4 (159.3 to 315.4)
Democratic Republic of the Congo	225.2 (190.9 to 296.7)	16.0 (5.3 to 31.7)	0.0 (0.0 to 0.0)
Djibouti	4.3 (3.7 to 5.3)	1.5 (1.0 to 2.2)	3.2 (2.1 to 4.6)
Dominica	0.5 (0.4 to 0.7)	0.3 (0.2 to 0.5)	0.5 (0.3 to 0.8)
Dominican Republic	161.4 (111.2 to 243.9)	58.1 (38.0 to 85.4)	284.6 (186.3 to 418.5)
Ecuador	39.6 (28.2 to 54.6)	31.6 (21.6 to 45.0)	144.5 (98.8 to 206.2)
Egypt	91.5 (55.4 to 148.6)	82.8 (50.8 to 125.2)	0.0 (0.0 to 0.0)
El Salvador	123.2 (96.4 to 159.8)	102.8 (80.6 to 129.7)	0.0 (0.0 to 0.0)
Equatorial Guinea	21.5 (10.1 to 42.1)	19.4 (9.7 to 33.8)	25.2 (12.6 to 43.9)
Eritrea	17.8 (16.8 to 19.7)	2.2 (1.3 to 3.1)	2.0 (1.2 to 2.9)
Ethiopia	783.8 (732.2 to 869.7)	90.4 (40.0 to 174.5)	316.4 (139.9 to 611.0)
Federated States of Micronesia	0.3 (0.2 to 0.4)	0.2 (0.1 to 0.3)	0.0 (0.0 to 0.0)
Fiji	1.2 (0.7 to 2.0)	1.0 (0.7 to 1.5)	0.0 (0.0 to 0.0)
Gabon	39.9 (21.9 to 75.4)	32.8 (21.1 to 45.7)	3.5 (2.2 to 4.9)
Georgia	41.1 (34.2 to 50.9)	21.7 (16.3 to 28.6)	4.3 (3.2 to 5.7)
Ghana	274.4 (169.5 to 526.3)	73.2 (21.2 to 154.7)	55.2 (16.0 to 116.6)
Grenada	0.6 (0.5 to 0.9)	0.4 (0.3 to 0.5)	0.6 (0.4 to 0.8)
Guatemala	100.0 (74.9 to 132.2)	69.3 (48.7 to 97.2)	44.5 (31.2 to 62.3)
Guinea	82.3 (74.6 to 96.9)	9.1 (4.6 to 15.8)	13.2 (6.6 to 23.0)
Guinea-Bissau	11.8 (10.5 to 14.5)	2.2 (0.9 to 4.5)	2.0 (0.8 to 4.2)
Guyana	17.8 (13.8 to 24.1)	7.2 (4.1 to 11.8)	7.4 (4.2 to 12.1)
Haiti	237.0 (233.2 to 242.7)	8.3 (4.9 to 12.6)	11.1 (6.6 to 16.9)
Honduras	53.2 (40.2 to 68.9)	31.0 (19.9 to 45.8)	0.0 (0.0 to 0.0)
India	1858.2 (1323.6 to 2655.6)	1276.4 (846.5 to 1895.0)	1416.6 (939.5 to 2103.0)
Indonesia	422.3 (351.6 to 517.5)	280.3 (209.6 to 375.3)	713.4 (533.7 to 955.4)
Iran	404.2 (233.6 to 651.1)	383.6 (216.9 to 622.1)	0.0 (0.0 to 0.0)

Iraq	21.8 (12.1 to 38.7)	16.8 (10.3 to 26.0)	2.2 (1.4 to 3.5)
Jamaica	43.6 (32.4 to 63.0)	17.6 (9.4 to 30.2)	50.6 (27.0 to 86.8)
Jordan	4.2 (2.8 to 6.3)	2.7 (1.5 to 4.6)	10.4 (5.7 to 18.0)
Kazakhstan	72.5 (50.1 to 109.1)	62.0 (39.3 to 98.7)	0.0 (0.0 to 0.0)
Kenya	1556.1 (1289.9 to 2038.6)	299.1 (169.3 to 478.4)	60.7 (34.4 to 97.1)
Kiribati	0.1 (0.1 to 0.2)	0.1 (0.0 to 0.1)	0.0 (0.0 to 0.0)
Kyrgyzstan	53.2 (48.2 to 59.7)	16.4 (12.3 to 22.7)	0.0 (0.0 to 0.0)
Laos	20.4 (18.3 to 23.8)	4.1 (2.1 to 7.4)	3.9 (1.9 to 6.9)
Lebanon	16.5 (9.3 to 27.1)	15.5 (8.9 to 24.5)	17.9 (10.2 to 28.2)
Lesotho	215.7 (176.7 to 266.8)	78.9 (40.0 to 129.8)	3.0 (1.5 to 5.0)
Liberia	27.0 (22.8 to 33.8)	3.3 (1.0 to 6.9)	0.6 (0.2 to 1.3)
Libya	6.6 (3.7 to 10.9)	6.1 (3.4 to 10.0)	6.7 (3.8 to 11.0)
Macedonia	8.8 (7.0 to 10.9)	5.9 (4.1 to 7.9)	0.5 (0.4 to 0.7)
Madagascar	18.9 (14.9 to 26.6)	6.9 (3.0 to 14.7)	0.0 (0.0 to 0.0)
Malawi	730.3 (665.8 to 831.4)	91.4 (49.2 to 153.0)	0.0 (0.0 to 0.0)
Malaysia	164.9 (128.7 to 204.9)	157.2 (121.7 to 196.6)	201.0 (155.6 to 251.3)
Maldives	2.4 (1.9 to 3.2)	1.4 (0.9 to 2.2)	1.8 (1.2 to 2.8)
Mali	86.9 (77.0 to 102.9)	20.2 (11.6 to 32.0)	0.0 (0.0 to 0.0)
Marshall Islands	0.2 (0.1 to 0.4)	0.2 (0.1 to 0.3)	0.0 (0.0 to 0.0)
Mauritania	12.1 (10.1 to 14.6)	4.2 (2.4 to 6.7)	5.6 (3.2 to 8.9)
Mauritius	20.7 (14.3 to 29.9)	17.7 (11.5 to 26.7)	9.4 (6.2 to 14.3)
Mexico	1140.3 (836.6 to 1551.2)	904.3 (627.0 to 1296.5)	625.2 (433.5 to 896.4)
Moldova	20.3 (16.0 to 27.2)	9.5 (5.5 to 16.5)	11.8 (6.8 to 20.5)
Mongolia	8.9 (7.2 to 11.3)	3.6 (2.4 to 5.5)	0.3 (0.2 to 0.5)
Montenegro	3.9 (2.6 to 5.6)	3.9 (2.6 to 5.5)	0.0 (0.0 to 0.0)
Morocco	42.8 (35.6 to 53.5)	23.5 (18.0 to 31.2)	22.7 (17.4 to 30.2)
Mozambique	1070.4 (1042.7 to 1123.0)	61.7 (33.6 to 115.0)	190.2 (103.7 to 354.5)
Myanmar	385.8 (370.9 to 405.8)	25.5 (12.6 to 43.9)	170.6 (84.4 to 294.1)
Namibia	413.3 (306.7 to 581.7)	267.2 (178.0 to 391.0)	0.0 (0.0 to 0.0)
Nepal	29.8 (22.6 to 46.8)	4.5 (1.5 to 9.5)	12.5 (4.1 to 26.1)
Nicaragua	99.6 (84.5 to 117.2)	53.1 (39.8 to 67.4)	60.6 (45.4 to 76.8)
Niger	24.9 (21.8 to 31.1)	4.0 (1.6 to 8.5)	3.9 (1.6 to 8.3)
Nigeria	1008.1 (921.3 to 1155.4)	160.7 (79.4 to 293.3)	652.2 (322.1 to 1190.1)
North Korea	2.3 (1.8 to 2.9)	1.9 (1.7 to 2.2)	2.0 (1.7 to 2.2)
Pakistan	79.6 (61.0 to 120.2)	38.3 (19.8 to 79.2)	16.0 (8.3 to 33.1)
Palestine	1.0 (0.7 to 1.5)	0.9 (0.7 to 1.1)	0.0 (0.0 to 0.0)
Panama	83.4 (53.0 to 123.3)	73.4 (47.8 to 109.9)	77.2 (50.3 to 115.7)
Papua New Guinea	84.5 (77.1 to 94.5)	17.9 (10.7 to 27.9)	0.0 (0.0 to 0.0)
Paraguay	31.9 (19.6 to 51.0)	18.4 (10.2 to 30.6)	49.8 (27.5 to 82.7)
Peru	130.5 (70.3 to 233.0)	114.6 (60.8 to 218.1)	171.4 (91.0 to 326.2)
Philippines	31.2 (22.5 to 43.1)	25.5 (18.2 to 35.9)	114.2 (81.6 to 160.8)

Romania	206.7 (151.0 to 273.7)	205.9 (150.3 to 272.7)	0.0 (0.0 to 0.0)
Russian Federation	1269.0 (719.5 to 2120.4)	1258.2 (709.0 to 2113.2)	1862.5 (1049.5 to 3128.1)
Rwanda	392.5 (352.5 to 450.3)	88.6 (49.2 to 147.6)	0.0 (0.0 to 0.0)
Saint Lucia	0.9 (0.6 to 1.2)	0.6 (0.4 to 0.7)	1.2 (0.8 to 1.6)
Saint Vincent and the Grenadines	1.2 (0.8 to 1.9)	0.9 (0.5 to 1.7)	0.0 (0.0 to 0.0)
Samoa	0.8 (0.6 to 1.1)	0.4 (0.3 to 0.6)	0.1 (0.1 to 0.1)
Sao Tome and Principe	0.8 (0.7 to 1.0)	0.2 (0.1 to 0.4)	0.0 (0.0 to 0.0)
Senegal	53.8 (46.9 to 65.5)	12.5 (5.0 to 25.2)	17.5 (7.0 to 35.1)
Serbia	21.7 (15.8 to 30.1)	21.3 (15.4 to 29.7)	52.9 (38.1 to 73.5)
Sierra Leone	49.4 (48.1 to 51.4)	2.3 (1.1 to 4.2)	11.0 (5.3 to 20.1)
Solomon Islands	0.2 (0.2 to 0.4)	0.2 (0.1 to 0.3)	0.0 (0.0 to 0.0)
Somalia	1.1 (0.9 to 1.6)	0.1 (0.1 to 0.2)	0.0 (0.0 to 0.0)
South Africa	4968.5 (3061.3 to 8059.8)	3728.4 (1995.2 to 6435.0)	2620.0 (1402.1 to 4522.0)
South Sudan	70.6 (67.6 to 78.0)	4.2 (3.1 to 5.3)	86.0 (63.4 to 108.1)
Sri Lanka	24.5 (18.1 to 33.9)	13.2 (7.2 to 22.2)	50.0 (27.2 to 84.1)
Sudan	41.4 (36.7 to 48.3)	7.0 (3.4 to 12.8)	11.8 (5.7 to 21.4)
Suriname	5.6 (3.9 to 7.7)	5.1 (3.7 to 6.9)	2.6 (1.9 to 3.5)
Swaziland	337.5 (263.7 to 446.6)	147.6 (77.4 to 250.2)	0.0 (0.0 to 0.0)
Syria	32.0 (24.1 to 43.6)	19.5 (11.8 to 30.4)	42.8 (25.9 to 66.5)
Tajikistan	55.7 (51.9 to 60.8)	13.8 (10.3 to 18.8)	0.0 (0.0 to 0.0)
Tanzania	1431.7 (1373.9 to 1532.5)	132.6 (87.3 to 188.6)	190.4 (125.3 to 270.9)
Thailand	1057.3 (859.0 to 1279.0)	1041.0 (844.3 to 1258.4)	0.0 (0.0 to 0.0)
The Gambia	16.8 (16.2 to 18.0)	1.8 (1.2 to 3.0)	0.7 (0.4 to 1.1)
Timor-Leste	7.2 (5.8 to 9.3)	3.1 (1.7 to 5.1)	4.9 (2.7 to 8.0)
Togo	64.6 (54.1 to 83.2)	11.7 (5.8 to 21.1)	0.0 (0.0 to 0.0)
Tonga	0.2 (0.1 to 0.2)	0.1 (0.1 to 0.1)	0.0 (0.0 to 0.0)
Tunisia	27.7 (21.5 to 37.3)	17.4 (10.3 to 28.1)	13.5 (8.0 to 21.8)
Turkey	432.6 (237.4 to 757.4)	429.4 (237.1 to 743.7)	0.0 (0.0 to 0.0)
Turkmenistan	25.0 (16.1 to 38.1)	21.5 (14.2 to 32.4)	0.0 (0.0 to 0.0)
Uganda	1604.5 (1449.1 to 1854.1)	123.2 (37.5 to 270.9)	47.2 (14.4 to 103.9)
Ukraine	364.3 (280.3 to 485.6)	170.2 (99.6 to 272.2)	354.7 (207.5 to 567.3)
Uzbekistan	113.0 (94.1 to 144.5)	47.7 (29.8 to 79.9)	25.9 (16.2 to 43.4)
Vanuatu	0.1 (0.1 to 0.2)	0.1 (0.0 to 0.1)	0.0 (0.0 to 0.0)
Venezuela	454.0 (289.8 to 678.3)	423.7 (267.4 to 641.3)	0.0 (0.0 to 0.0)
Vietnam	310.1 (252.8 to 401.2)	68.5 (34.3 to 118.9)	453.1 (226.8 to 785.7)
Yemen	5.4 (2.9 to 9.8)	1.5 (0.9 to 2.0)	0.0 (0.0 to 0.0)
Zambia	781.1 (731.9 to 866.5)	89.4 (41.2 to 173.0)	307.6 (142.0 to 595.4)
Zimbabwe	486.5 (438.0 to 567.0)	80.9 (43.2 to 136.1)	146.3 (78.2 to 246.2)

Work cited

1 World Development Indicators (WDI) | Data Catalog. https://datacatalog.worldbank.org/dataset/world-development-indicators (accessed Jan 31, 2019).

2 World Economic Outlook Database October 2018. https://www.imf.org/external/pubs/ft/weo/2018/02/weodata/index.aspx (accessed Jan 31, 2019).

3 Robert C. Feenstra RI. Penn World Table 9.0. 2016. DOI:10.15141/S5J01T.

4 National Accounts - Analysis of Main Aggregates (AMA). https://unstats.un.org/unsd/snaama/Index (accessed Jan 31, 2019).

5 WHO | Global Health Observatory (GHO) data. WHO. http://www.who.int/gho/en/ (accessed Jan 31, 2019).

6 Global Burden of Disease Health Financing Collaborator Network. Past, present and future of global health financing: a review of country spending on health and development assistance for 195 countries, 1995–2050. Lancet 2019; published online April 25. DOI:10.1016/S0140-6736(19)30841-4.

7 Gakidou E, Afshin A, Abajobir AA, *et al.* Global, regional, and national comparative risk assessment of 84 behavioural, environmental and occupational, and metabolic risks or clusters of risks, 1990–2016: a systematic analysis for the Global Burden of Disease Study 2016. *The Lancet* 2017; **390**: 1345–422.

8 Dicker D, Nguyen G, Abate D, *et al.* Global, regional, and national age-sex-specific mortality and life expectancy, 1950–2017: a systematic analysis for the Global Burden of Disease Study 2017. *The Lancet* 2018; **392**: 1684–735.

9 James SL, Abate D, Abate KH, *et al.* Global, regional, and national incidence, prevalence, and years lived with disability for 354 diseases and injuries for 195 countries and territories, 1990–2017: a systematic analysis for the Global Burden of Disease Study 2017. *The Lancet* 2018; **392**: 1789–858.

10 Hollingsworth B, Peacock S. Efficiency Measurement in Health and Health Care. Oxon: Routledge.

11 Remme M, Siapka M, Sterck O, Ncube M, Watts C, Vassall A. Financing the HIV response in sub-Saharan Africa from domestic sources: Moving beyond a normative approach. *Soc Sci Med 1982* 2016; **169**: 66–76.
